# Placental Microarray Profiling Reveals Common mRNA and lncRNA Expression Patterns in Preeclampsia and Intrauterine Growth Restriction

**DOI:** 10.3390/ijms21103597

**Published:** 2020-05-20

**Authors:** Diana Medina-Bastidas, Mario Guzmán-Huerta, Hector Borboa-Olivares, César Ruiz-Cruz, Sandra Parra-Hernández, Arturo Flores-Pliego, Ivan Salido-Guadarrama, Lisbeth Camargo-Marín, Eliakym Arambula-Meraz, Guadalupe Estrada-Gutierrez

**Affiliations:** 1 Unidad de Investigación en Reproducción Humana, Instituto Nacional de Perinatología-Facultad de Química, Universidad Nacional Autónoma de México, Mexico City 11000, Mexico; dianameedinab@gmail.com; 2 Departamento de Medicina Traslacional, Instituto Nacional de Perinatología, Mexico City 11000, Mexico; mguzmanhuerta@yahoo.com.mx (M.G.-H.); lisbethcamargo@yahoo.com.mx (L.C.-M.); 3 Subdirección de Investigación en Intervenciones Comunitarias, Instituto Nacional de Perinatología, Mexico City 11000, Mexico; h_borboa1@yahoo.com; 4 Hospital de Ginecología y Obstetricia No. 4, Luis Castelazo Ayala, Instituto Mexicano del Seguro Social, Mexico City 01090, Mexico; ginecol.2011@gmail.com; 5 Laboratorio de Inmunobioquímica, Instituto Nacional de Perinatología, Mexico City 11000, Mexico; sandrabpahdz@gmail.com (S.P.-H.); arturo_fpliego@yahoo.com.mx (A.F.-P.); 6 Laboratorio de Biología Computacional, Instituto Nacional de Enfermedades Respiratorias Ismael Cosío Villegas, Mexico City 14080, Mexico; silvervann@gmail.com; 7 Laboratorio de Genética y Biología Molecular, Facultad de Ciencias Químico Biológicas, Universidad Autónoma de Sinaloa, Culiacan 80040, Mexico; eliakymarambula@hotmail.com; 8 Dirección de Investigación, Instituto Nacional de Perinatología, Mexico City 11000, Mexic

**Keywords:** preeclampsia, intrauterine growth restriction, placenta, microarray, gene expression, LncRNA

## Abstract

Preeclampsia (PE) and Intrauterine Growth Restriction (IUGR) are major contributors to perinatal morbidity and mortality. These pregnancy disorders are associated with placental dysfunction and share similar pathophysiological features. The aim of this study was to compare the placental gene expression profiles including mRNA and lncRNAs from pregnant women from four study groups: PE, IUGR, PE-IUGR, and normal pregnancy (NP). Gene expression microarray analysis was performed on placental tissue obtained at delivery and results were validated using RTq-PCR. Differential gene expression analysis revealed that the largest transcript variation was observed in the IUGR samples compared to NP (*n* = 461; 314 mRNAs: 252 up-regulated and 62 down-regulated; 133 lncRNAs: 36 up-regulated and 98 down-regulated). We also detected a group of differentially expressed transcripts shared between the PE and IUGR samples compared to NP (*n* = 39), including 9 lncRNAs with a high correlation degree (*p* < 0.05). Functional enrichment of these shared transcripts showed that cytokine signaling pathways, protein modification, and regulation of JAK-STAT cascade are over-represented in both placental ischemic diseases. These findings contribute to the molecular characterization of placental ischemia showing common epigenetic regulation implicated in the pathophysiology of PE and IUGR.

## 1. Introduction

Preeclampsia (PE) and Intrauterine Growth Restriction (IUGR) are two of the great obstetrical syndromes that are significant contributors to maternal and perinatal morbidity and mortality [1]. These pregnancy disorders carry severe health consequences for both mother and fetus, particularly when they manifest together [2]. PE is characterized by de novo hypertension in pregnancy and proteinuria (≥3 g per 24 h); in the absence of proteinuria, diagnosis is established when de novo hypertension is associated with thrombocytopenia, renal insufficiency, impaired liver function, pulmonary edema or new-onset cerebral or visual disturbances [3,4]. On the other hand, IUGR is defined as a failure of the fetus to achieve its genetic growth potential, usually diagnosed by the statistical deviation of fetal size within the population-based standard growth curve in combination with hemodynamic alterations in the fetoplacental circulation [1,5,6].

PE and IUGR are considered placental ischemic diseases that share a common background where a defective placentation process and an incomplete remodeling of the spiral arteries due to reduced trophoblast invasion lead to placental insufficiency. The placenta is a transient organ that fulfills key tasks to ensure nourishment and oxygen diffusion for the developing fetus. It also works as a waste filtration system and as a fetal protective barrier. Moreover, the placenta performs other functions to guarantee physiological adaptations of mother and fetus, providing endocrine and immune support during pregnancy. In placental ischemic diseases, the impaired capacity of the trophoblast to invade uterine spiral arteries causes blood flow resistance, placental damage, hypoxia, acute atherosis, immune modifications and oxidative stress that lead to impaired cell function, release of proinflammatory cytokines, selective suppression of protein synthesis in the endoplasmic reticulum and apoptosis. Although the question about why substantial maternal systemic effects are limited to PE and do not appear in other placental ischemic disorders is not fully answered, evidence suggests that the higher levels of oxidative stress along with the severe proinflammatory environment as well as abnormal maternal vasculature lead to endothelial dysfunction, the hallmark of PE [5,7,8].

Research focused on the study of placental circulation, metabolism, placental phenotyping, and transcriptome analysis has successfully evidenced the relationship between these pregnancy disorders and impaired placental function. Particularly, transcriptome analysis either using RNA-seq or microarray techniques has been used to elucidate possible molecular mechanisms underlying the pathophysiology and even identify placental derived biomarkers that could refine the prediction and diagnosis of PE and IUGR [9,10]. While most studies have focused on mRNA, long non-coding RNAs (lncRNAs) have recently come into play in placental research focusing on their role in pregnancy complications and showing that altered expression on several lncRNAs is linked to a number of placental disorders, emerging as potential regulators of multiple molecular pathways involved in the pathogenesis of placental diseases [11]. PE is the placental pathology most frequently studied and represents the larger part of the transcriptomes data sets available [12,13,14,15,16,17,18], while other pregnancy complications of placental origin as IUGR have been less explored, therefore there is still a gap to be filled regarding the transcriptional signature shared among them as well as in the possibility to identify candidate biomarkers for IUGR. Thus, the present study aims to compare the placental gene expression profiles including mRNA and lncRNAs from pregnant women who have undergone PE, IUGR, or both.

## 2. Results

### 2.1. Clinical, Anthropometric, and Placental Histopathological Parameters of the Study Groups

Forty-five pregnant women with intact membranes, no clinical evidence of intrauterine infection, singleton pregnancy, and who delivered by cesarean section (C-section) with no evidence of active labor were included in the study and divided into four study groups: intrauterine growth restriction (IUGR, *n* = 12), preeclampsia with severe features (PE, *n* = 11), preeclampsia with intrauterine growth restriction (PE-IUGR, *n* = 11) and normal pregnancy (NP, *n* = 11), this last group was constituted by women with uncomplicated pregnancies at term who delivered normal weight newborns. Disease diagnosis was performed according to the 2018 ACOG criteria. After enrolment, follow up was carried out until six weeks postpartum to discard the development of hypertensive disorders in the NP and IUGR groups. Clinical and anthropometric data from the study groups are represented in Table 1. No differences in maternal age, Pregestational Body Mass Index (pBMI), Gestational Weight gain (GWG), and platelet count were observed between the study groups. Laboratory tests for 24 h urine protein, urea, creatinine, and uric acid as well as blood pressure measurements are in accordance with the diagnosis of PE and PE-IUGR groups. Moreover, all IUGR were in the ≤ 3rd Hadlock percentile at the moment of diagnosis and presented abnormal Doppler waveforms in the uterine, umbilical, and/or middle cerebral artery in the last ultrasound.

Nulliparity among women was significantly higher in the PE-IUGR group compared to NP (Table 1, *p* < 0.05), and weight and length of newborns, as well as gestational age at birth, was significantly different in all study groups compared to NP as expected. 

Additionally, placental histopathology reports for the IUGR, PE as well as for the PE-IUGR groups, demonstrated signs of placental insufficiency with the presence of placental infarction, distal villous hypoplasia, placental villous hypermaturation, syncytial knots, and/or massive perivillous fibrin deposition (Table S1). No differences were found in the frequency of the histopathological findings between any of the pathologies.

### 2.2. Global Placental Gene Expression Profiles in Preeclampsia and Intrauterine Growth Restriction

**Figure 1:**
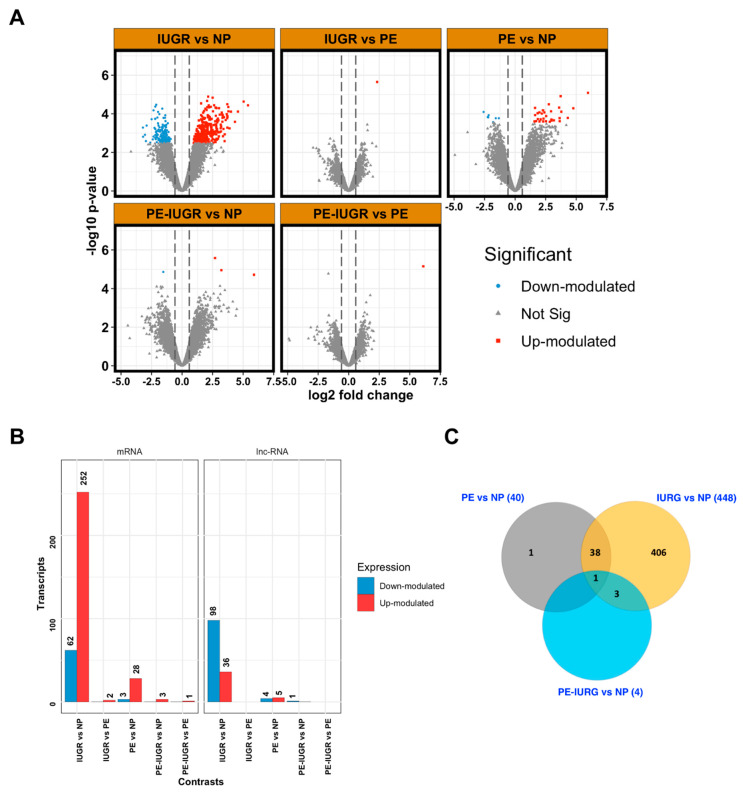
Distribution of differentially expressed genes in different comparisons between clinical groups. (**A**) Volcano plots show the differentially expressed genes for any of the comparisons made between different clinical groups. The *y*-axis indicates the statistical significance expressed as the-log10 of the *p*-values and the *x*-axis shows the rate of expression change between experimental groups in log2 base (log2 Fold change). Red dots indicate significantly over-expressed genes (with a log2 Fold change > 1.5 and adjusted *p* value < 0.1) and blue dots indicate significantly under-expressed genes (with a log2 Fold change < −1.5 and adjusted *p* value < 0.1). (**B**) The number of over- and under-expressed genes divided by transcript type are represented vertically in each comparison between groups representing different clinical conditions as indicated. (**C**) Venn diagram displaying the number of transcripts that show similar significant differential expression in different comparisons between groups. PE, preeclampsia; IUGR, Intrauterine Growth Restriction; PE-IUGR, Preeclampsia with Intrauterine Growth Restriction; NP, Normal Pregnancy.

Microarray data were collected from 29 samples, representing the different clinical conditions defined in our study (Table 1, a). A total of 463 transcripts were differentially expressed at a significant level in all pair-wise comparisons between clinical conditions. We detected the greatest variation in gene expression in the IUGR group compared to NP, observing 461 differentially expressed genes (DEG). Out of these, 314 mRNAs (252 up-regulated and 62 down-regulated) and 133 lncRNAs (36 up-regulated and 98 down-regulated) (Figure 1A; Table S2).

Moreover, when PE was compared against NP, a total of 40 differentially expressed genes emerged, including 31 mRNAs (28 up-regulated and 3 down-regulated) and 9 lncRNAs (up-regulated and 4 down-regulated) (Figure 1A; Table S2), and our analysis was able to detect four transcripts differentially expressed between PE-IUGR and NP.

Next, we decided to focus on better characterizing those genes that showed differential expression between two or more pair-wise comparisons, as these transcripts may be more informative biomarkers, with greater utility at distinguishing placental diseases. Of interest, we found 42 genes that showed significant expression changes that overlapped between IUGR and PE (Figure 1A). As expected, 39 genes out of the initial 42 (90.7%) showed expression changes both in IUGR and PE groups compared to NP, which constitute 97.5% of the 40 differentially expressed transcripts between PE and NP.

**Figure 2:**
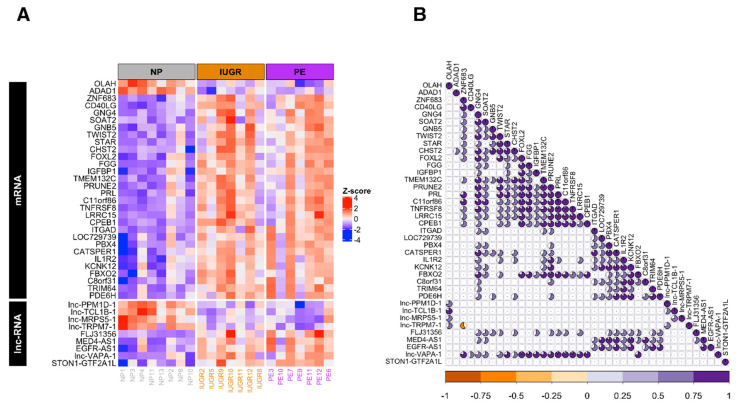
Selected genes differentially expressed in Intrauterine Growth Restriction and Preeclampsia compared to Normal Pregnancy. (**A**) Heatmap shows the expression levels of 39 selected genes (in rows), that are differentially expressed in IUGR and PE conditions, across samples (in columns) grouped by clinical condition. Expression levels are presented as z-score values for the purpose of visualizing expression differences across samples. The color palette indicates higher expression values in red shades and lower ones in blue shades. (**B**) Pair-wise correlations based on expression values were computed for each pair of genes in the matrix shown in A. Pearson coefficient computed for each pair of genes are presented in a triangular matrix, where genes on rows are projected diagonally and statistically significant (*p* value < 0.05) Pearson coefficients of the respective comparisons against the rest of genes are shown in a pie chart manner, that is, the magnitude of Pearson correlation. Positive correlations are indicated in purple shades and negative ones in orange shades.

Then we inspected the expression levels of these 39 genes across IUGR, PE, and NP samples and evaluated their degree of correlation across IUGR and PE samples. In concordance with general results, the majority of these selected genes were over-expressed in both conditions (Figure 2A). Moreover, at examining the statistically significant pair-wise correlation (*p-*value < 0.05 for Pearson coefficient), we detected two clusters of mostly positive correlated genes. The first cluster included the mRNAs *STAR, CHST2, FOXL2, FGG, IGFBP1, TMEM132, PRUNE2, TNFRSF1 CPEB1,* and the lnc-RNAs *MED4-AS1* and *EGFR-AS1*, while the second cluster was mostly composed of mRNAs *FBXO2*, *KCNK12, IL1R2,* and *CATSPER1* mRNAs and *lnc-VAPA-1* (Figure 2B). The pattern that emerged from this analysis could be indicative that these genes are undergoing a common regulation process in both conditions.

### 2.3. Gene Ontology Analysis for the Differentially Expressed Genes in IUGR and PE

To explore the biological and molecular relevance of the DEG, we first performed an over-representation analysis of KEGG in the IUGR group (Table 2, Table S3). The analysis showed that the set of down-regulated genes were mostly enriched in neuroactive ligand-receptor interaction, PI3K-Akt signaling pathway, and fatty acid biosynthesis (*p* < 0.05). On the other hand, the up-regulated genes in IUGR were mainly enriched in immunological processes including cytokine-cytokine receptor interaction, chemokine signaling pathway, and primary immunodeficiency, as well as allograft rejection and graft-versus-host disease, among others.

**Figure 3:**
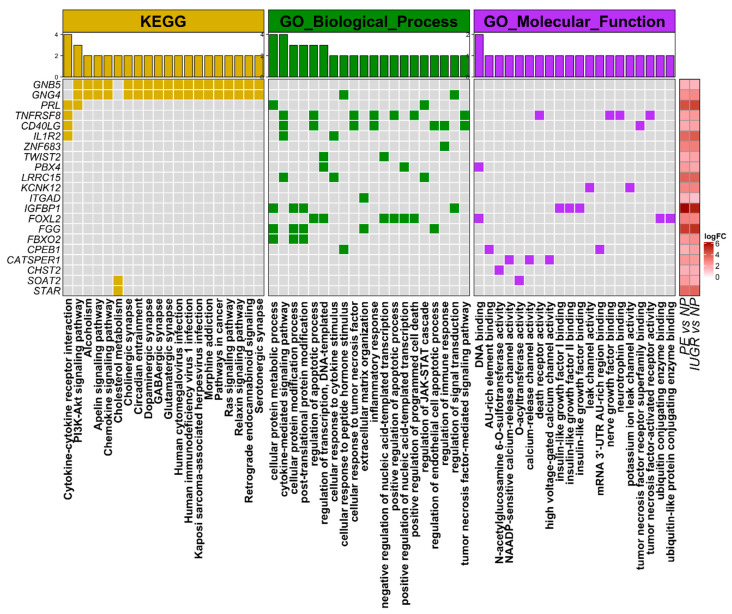
Biological and pathological processed over-represented in 39 differentially expressed genes. In the central panel, matrix shows the genes that are part of the list over-represented terms from KEGG, GO_Biological_processes, and GO_molecular function annotations. Bar plot on the top panel shows the total number of selected genes for each biological term. On the right, matrix shows the level of log2-base expression change (log2 fold-change) for each of the selected differentially expressed genes in the indicated contrast.

Next, we focus on exploring KEGG, GO_Biological_Process, and GO_Molecular_function terms represented among the 39 mRNAs similarly regulated in IUGR and PE. Thus, we could identify related biological and molecular processes and get further insights into the mechanisms involved in placental diseases. We found a predominant representation of GO_Biological_Process terms among the selected genes, such as cytokine signaling pathways, protein modification, and regulation of JAK-STAT cascade, among others (Figure 3). Similarly, we found that *GNB5*, *GNG4*, *PRL*, *TNFRSF8, CATSPER1*, *CD40LG, IL1R2, CHST2*, *CPEB1*, *FBXO2*, *FGG*, *FOXL2*, *IGFBP1*, *LRRC15*, *KCNK12, ITGAD*, *PBX4*, *SOAT2*, *STAR*, *TWIST2*, and *ZNF683* are the most prevalent genes, appearing among the top-20 over-represented terms (Figure 3).

### 2.4. qPCR Validation for Target Genes in Preeclampsia and Intrauterine Growth Restriction

**Figure 4:**
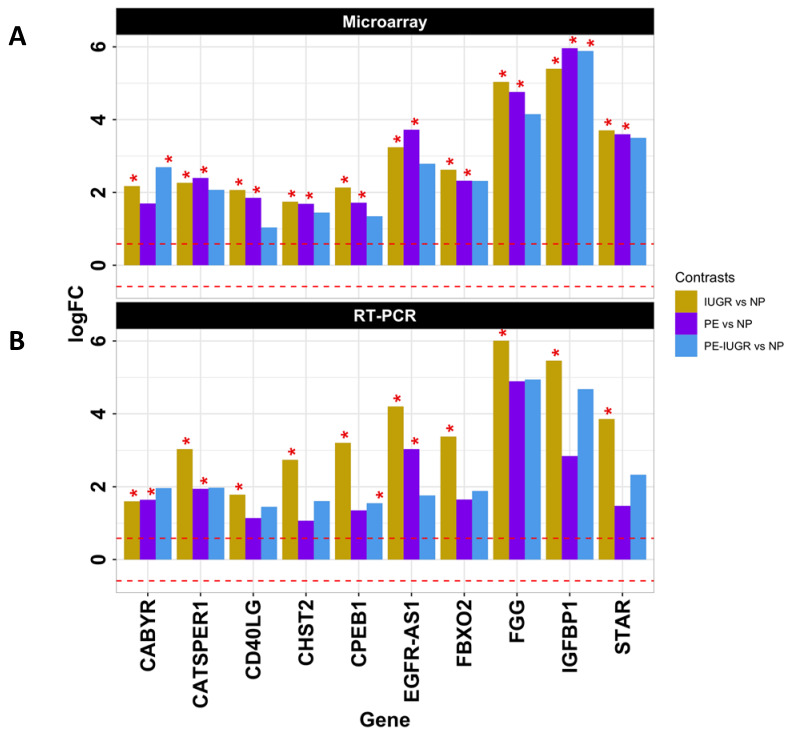
Validation of selected differentially expressed genes. Genes with the highest log2 fold-change and/or involvement in top-20 biological, molecular, or pathological processes were selected, and their expression change was evaluated independently by an orthogonal RT-PCR method. (**A**) Bar plot represents log2 fold-changes results from microarray data, plotted on the *y*-axis, for each gene, here depicted horizontally. The asterisks indicate where change was statistically significant (i.e., FDR < 0.1) and the horizontal dashed red lines marks the fold-change threshold considered as significant (−1.5 < fold-change > 1.5. (**B**) Bar plot represents delta-delta-Ct (-DDCT) results from RT-PCR data, plotted on the *y*-axis, for each gene, here depicted horizontally. The asterisks indicate where change was statistically significant (i.e., *p*-value < 0.05) and the horizontal dashed red lines marks the fold-change threshold considered as significant.

In order to confirm the most important changes in expression among these 43 genes, we decided to restrict the list of candidate genes for validation based on the following criteria: (i) level of expression change and overlap degree between any pair-wise comparisons, (ii) statistical significance in the contrasts they appeared, and (iii) their presence and number of over-represented biological and molecular processes. Through these criteria, we selected 9 protein-coding genes including *IGFBP1*, *FGG, FBXO2*, *CPEB1*, *CHST2*, *CD40LG*, *CATSPER1*, *CABYR,* and *STAR* and the lncRNA *EGFR-AS1* (Figure 4A, Figure S1). Gene expression analysis was performed in placental tissue from 12 IUGR, 11 PE, 11 PE-IUGR, and 11 NP (Table 1, b). Results showed significant over-expression (*p <* 0.05) of the selected protein coding genes and the lncRNA *EGFR-AS1* in IUGR samples compared to the NP group. Moreover, PE samples showed up-regulation for *CABYR*, *CATSPER1,* and EGFR-AS1. We also observed that *CPEB1* and *IGFBP1* are both up-regulated in the PE-IUGR group compared to NP (Figure 4, Figure S1).

## 3. Discussion

In this study, we assessed and compared the placental expression profiles of pregnancies complicated by IUGR, PE, and PE compound with IUGR. These disorders are within the spectrum of placental ischemic disease and represent two of the main causes of perinatal morbidity and mortality [19]; moreover, mothers and fetuses affected with these pathologies are at higher risk of metabolic and cardiovascular diseases later in life [1,20,21,22]. The placenta is the primary interface between fetus and mother, and its main function relies on delivering nutrients and oxygen to the fetus. This transition organ is also in charge of several physiological functions including modulation of the mother’s immune system by promoting tolerance and preventing immunological rejection and acts as an endocrine organ producing several important hormones during pregnancy [23,24,25,26].

Robust evidence has shown that major changes in placental histopathology, cellular and molecular function, and in gene expression are involved in the pathophysiology of both PE and IUGR [10,27,28,29]. Over the past decade, omic sciences that are aimed to epigenetics, transcriptomics, GWAS (Genome-wide associations), proteomics, and metabolomics have allowed to further characterize the molecular mechanisms underlying these conditions, and several placental genes such as *VEGF*, s*Flt1*, *ENG, EDN1, IGFBP1,* and *LEP* have been identified on different clinical PE and/or IUGR phenotypes [13,14,18,30,31,32,33,34,35]. Moreover, the use of advanced bioinformatics on aggregated data sets has led to new molecular classifications and helped to elucidate the transcriptional factors involved in the regulation of many genes associated with PE [17,36,37,38,39,40]. However, only a few studies have compared the gene expression profiles between PE and IUGR, and the particular contribution of lncRNAs for placental ischemic diseases is yet to be explored. 

All PE and IUGR cases included in our study showed clinical and histopathological evidence of placental dysfunction, and no differences were observed in confounding variables such as maternal age, pBMI, and GWG. Importantly, we only included samples obtained from pregnancies delivered by C-section to avoid alterations in gene expression induced by labor. However, one of the main limitations of our study relies on the differences in nulliparity, gestational age at birth, and disease onset from the PE-IUGR group. It is possible that these characteristics did not allow us to further explore the placental transcriptome from this group; particularly, disease onset and gestational age at birth may be responsible for transcriptional changes that are not comparable with placentas affected by late-onset disease or NP at term. Nonetheless, we decided to use normal pregnancies at term as a control group due to the fact that matching controls by gestational age imply using samples from premature deliveries exposed to different transcriptional variations that may be associated with other obstetric pathologies [41]. Regardless of the findings with respect to the PE-IUGR, we were able to detect extensive transcriptional changes in the placentas from IUGR samples as well as a crucial set of differentially expressed transcripts shared between PE and IUGR. 

Poor fetal growth has been linked to inherited genetic and epigenetic variations, as well as to alterations related to early implantation and placentation that are critical to maintain nutrient supply to the fetus [5,42,43]. It has also been proved through many human studies and animal models that IUGR is related to hypoxemia, affected placental transport capacity, alteration on vasculogenesis and angiogenesis, dysregulation of insulin-like growth factors activity, increased levels of apoptosis, autophagy, and glucocorticoid actions, as well as an increased in inflammatory response and imbalance of the immune system [6,44,45,46].

Our first finding was that the IUGR samples showed the greatest transcript variation, displaying down-regulation of genes involved in neuroactive ligand-receptor interaction, fatty acid biosynthesis, and pathways involving NOS3 activity, including arginine biosynthesis and metabolism, angiogenesis as well as the VEGF signaling pathway. Endothelial nitric oxide synthase 3 (eNOS3) takes part in placental angiogenesis and vasculogenesis and is highly expressed during embryonic and fetal development [47], and its low expression is directly related with lower nitric oxide availability, impaired trophoblast invasion and as a result, reduced uteroplacental blood flow and oxygen levels observed in IUGR [48]. Furthermore, it has been suggested that early endothelial dysfunction in individuals born after IUGR plays an important role in the subsequent development of hypertensive disease which could be related to a defective eNOS function and oxidative stress [49]. 

Contrastingly, up-regulated mRNAs in IUGR showed mainly pathways related to immunological processes such as cytokine–cytokine receptor interaction, T cell receptor signaling pathway, and allograft rejection. *ITK* (Il12 inducible T cell kinase), *ZAP70* (Zeta-chain-associated protein kinase 70), and *LCK* (LCK Proto-Oncogene, Src Family Tyrosine Kinase) gene dysregulation was highly involved in many of the immunological processes identified in our study. *LCK* and *ZAP70* are part of the T cell maturation and differentiation pathway [50] and have been identified in growth-restricted placentas as key genes associated with inflammatory processes underlying this pathology [51]. Src family kinases such as LCK play different roles on trophoblast function affecting trophoblast invasion and differentiation [52]. Thus, alteration in their expression could be associated with the inadequate transformation of the spiral arteries that leads to placental ischemia. 

Moreover, other placental microarray studies such as the one performed by Sitras et al. also reported differential expression on genes involved in inflammation-mediated by cytokines and chemokines related pathways [45]. Recently, Wang et al. demonstrated through whole transcriptome sequencing in IUGR umbilical cord that core regulatory networks involving inflammatory response, graft versus host disease, and allograft rejection are involved in this pathology, very similar to our findings in placenta [53]. In addition to immunological related pathways involved in IUGR, several studies have also highlighted the importance of gene dysregulation associated with apoptotic processes, degradation of noxious chemicals, angiogenesis, and hypoxia in growth restriction physiopathology [35,54,55]. Together, all this evidence provides a deeper insight into the gene signatures related to IUGR due to placental dysfunction and enriches the current knowledge regarding immunological theory surrounding IUGR.

On the other hand, although PE is the most studied placental pathology, several theories exist about the cause of this disorder and there is no consensus on its etiology [37], but a poor or impaired placentation process and maternal vascular endothelial dysfunction are known to be the hallmarks of this disease [28,56]. PE is a syndrome with a spectrum of phenotypes with different degrees of severity associated with the onset of the symptoms that may reflect on consequences to the fetus. Therefore, current clinical classification is based mainly on the clinical severity and the moment of clinical presentation: early-onset (<34 weeks) or late-onset (≥34 weeks) [57,58]. Moreover, PE with severe features is diagnosed when at least one of the following criteria is present; systolic blood pressure ≥ 160 mmHg and/or diastolic blood pressure of ≥ 110 mmHg, thrombocytopenia impaired liver function, renal insufficiency, pulmonary edema, new-onset headache unresponsive to medication, and visual disturbances [3].

Early-onset PE is associated with the most severe spectrum of the disease and the presentation of IUGR. This association appears to be related to a deeper failure in the placentation process at the earliest stages of pregnancy during the pre-clinical phase of PE that eventually leads to higher oxidative stress levels, maternal vascular endothelial dysfunction, placental hypoxia, and intrauterine growth restriction [7,59,60]. On the other hand, PE without IUGR is often associated with late-onset of the symptoms and the systemic effects seem to be related to maternal factors such as obesity, metabolic diseases and a genetic predisposition for cardiovascular disease that could induce maternal dysfunction in response to factors released from the placenta [34,61,62]. However, the answer to why the systemic effects of placental ischemia are limited to PE, and the diverse pathological pathways that lead to the different clinical scenarios of this disease are not fully established and are still a subject of intense investigation. Thus, the wide spectrum of clinical manifestations observed in PE results particularly challenging for researchers that aim to find its causes for early diagnosis and prevention [63]. On top of that, it has been accurately stated that PE is a disease of the mother, fetus, and placenta and that clinical manifestations are the result of an interplay between several factors and disease pathways [61,64]. 

Our second main finding resulted from the placental transcriptome characterization of PE cases which were all representative from the severe spectrum of the disease. First, we identified a total of 40 transcripts differentially expressed when compared to NP, including 9 lncRNAs, and it was observed that 97.5% were also present in the set of DEG in the IUGR as well that regulation of these transcripts was also in the same direction for both groups with a high correlation degree. We found that the identified transcripts are mainly involved in molecular pathways associated with placental ischemia. Other studies have also identified placental gene modules and signatures of placental malfunction: Sober et al. reported the analysis of DEG in late-onset PE (LO-PE), gestational diabetes, small for gestational age (SGA) and large for gestational age (LGA) placentas and detected the highest concordance in gene expression disturbances compared to NP between LO-PE, LGA, and SGA placentas [65]. Moreover, Gabor-Than et. al reported two major dysregulated placental diseases gene modules either associated to maternal pathways, where maternal factors such as systemic inflammatory changes later induce trophoblastic functional alteration that not necessarily affect fetal growth, or associated to genes related to direct placental pathways, where altered differentiation of the trophoblast leads to gene dysregulation that is more associated with early-onset PE. Importantly, the reported genes from this module are associated with fetal growth and metabolism [61]. In our study, the resulting enriched pathways from DEG shared between PE and IUGR also showed genes involved in metabolism such as *STAR* (Steroidogenic acute regulatory protein) and *SOAT2* (Sterol -Acyltransferase 2), which are implicated in cholesterol metabolism. Furthermore, we also detected a set of genes related to protein metabolic processes including *IGFBP1* (Insulin-Like Growth Factor Binding Protein 1), *FGG* (Fibrinogen Gamma Chain), *FBXO2* (F-Box Protein 2), and *PRL* (Prolactin).

Steroid hormone synthesis is vital for pregnancy maintenance and is highly regulated by the placenta. It has been suggested that maternal cardiovascular adaptation is mediated, in part, by primary estrogens, which are synthesized by the uteroplacental unit using circulating steroid precursors from both the maternal and fetal adrenal gland [66]. Additionally, evidence supports that impairment of placental steroidogenesis is associated with PE severity [67], and it is widely accepted that changes in the synthesis and metabolism of key placental nutrients and hormones influence the rate of fetal intrauterine growth [46,68,69,70].

STAR is a critical, rate-limiting regulator of steroid production and cholesterol intracellular trafficking in steroidogenic tissues. In mice, STAR is highly expressed in placental giant trophoblast cells during a limited time at mid-pregnancy [71,72]. *SOAT2* encodes for cholesterol acyltransferase 2 which is responsible for the synthesis of cholesteryl esters (CE) that constitute the lipid core of chylomicrons and very low-density lipoproteins (VLDL) [73]. Importantly, *SOAT2* has previously been identified in genetic regulatory networks associated with PE [74]. Thus, dysregulation of these protein-encoding genes reflects the importance of cholesterol synthesis and metabolism during the development of placental ischemia.

Our findings regarding the differential expression of genes such as *IGFBP1* and *PRL* as well as other genes also involved in the p13-akt-signaling pathway like *GNB5* and *GNG4,* highlight the involvement of these pathways in placental ischemia. These results are in accordance with previous findings reported by Rupasri et al., on a glucocorticoid-induced IUGR rat model, where it was demonstrated that placental insufficiency is associated with dysregulated placental PRL family gene expression and down-regulation of the IGF-II/Akt signaling pathway, which ultimately increases placental apoptosis [75]. Of note, *IGFBP1* which is likely to be more associated with IUGR was found to be upregulated in PE and IUGR. This protein-encoding gene is mainly associated with trophoblast implantation and invasion. Lowly expressed *IGFPB* during the first trimester of pregnancy has been implicated in implantation failure as well as impaired placentation which leads to the placental insufficiency observed in both conditions; moreover, robust evidence shows that this protein is highly expressed in term placentas from pregnancies complicated by PE or IUGR, therefore, research lines have aimed to study this protein as a potential biomarker in maternal serum from IUGR pregnancies. However, results are discrepant among studies and some suggest that maternal IGFBP1 serum measurement during the first trimester of pregnancy could help to predict growth restriction in a combined assessment with other biomarkers and ultrasound approaches [55,76,77,78,79].

Furthermore, our analysis from shared transcripts between PE and IUGR also showed dysregulation of genes involved in several immunologic responses such as cytokine-mediated signaling pathways, inflammatory response, and immune regulation, including *CD40L*, *TNFRSF8*, *IL1R2*, *LRRC15*, and *ZNF683*, as well as genes related to the JACK-STAT cascade such as *PRL* and *OLAH.* This is in accordance with a growing body of evidence for both PE and IUGR regarding the excessive inflammatory up-regulation and is now considered to be a signature for placental ischemia [19,80,81,82]. However, other reports have compared the transcriptional profiles between PE and IUGR or SGA pregnancies with results that are not fully comparable. Nishizawa et al. reported a set 62 DEG shared between PE and IUGR placentas, showing mainly up-regulation of anti-angiogenic factors [32], while Huang et al. performed a targeted in silico analysis from available PE and IUGR microarray data sets demonstrating the existence of a group of membrane transporters with altered gene expression in both conditions, which could be related with the impaired materno-fetal nutrient transference [83]. A similar bioinformatic approach, but on a larger cohort, was conducted by Gibbs et al., using unsupervised clustering on placental gene expression data from normotensive and hypertensive suspected fetal growth restriction; their findings showed that there could be at least two pathological causes of normotensive IUGR, and that there is a high degree of similarity between normotensive and hypertensive IUGR placentas. They also found that these IUGR subtypes are comparable to PE placental subtypes, showing common dysregulation patterns, and that it is possible to maintain a maternal normotensive state until term despite a highly affected placenta [40]. 

Furthermore, we also discovered a signature of lncRNAs shared in our studied pathologies. The set of transcripts included *lnc-PPM1D-1*, *lnc-TCL1B-1*, *lnc-MRPS5-1*, *lncTRPM7-1*, *MED4-AS1*, *EGFR-AS1*, *FLJ31356*, *lnc-VAPA-1*, *STON1-GTF2A1L*. LncRNAs make up a majority of the human transcriptome with fundamental regulatory roles in many physiological aspects including angiogenesis, apoptosis, cell proliferation, and migration, as well as inflammation and gametogenesis, and alteration in their function has been lately implied in both PE and IUGR. Moreover, lncRNAs regulate gene expression at the epigenetic, transcriptional, and post-transcriptional level. There are previous reports regarding differential lncRNA expression between PE and normal pregnancies [84,85,86] as well as for IUGR, compared to normal pregnancies [11,87,88]. However, as far as we know, this is the first work reporting a set of lncRNAs for both pregnancy complications; furthermore, this also reinforces the idea that there are underlying epigenetic mechanisms implicated in placental ischemic diseases that could be in part responsible for the molecular pathology and transcriptional changes observed in these conditions. Importantly, *EGFR-AS1* has been reported as an important target for PE pathology [89]. An in vitro study demonstrated that a knock-down of this transcript inhibits cell proliferation and that overexpression showed the opposite result. *EGFR-AS1* knock-down was also associated with decreased expression of p-JAK and p-STAT; thus, it appears that this lncRNA regulates the JAK-STAT signaling pathway [90]. Interestingly, our gene ontology analysis also this pathway appears to be up-regulated not only for PE but also for IUGR. This is an example of the mechanisms in which lncRNAs are modulating important molecular pathways implicated in placental ischemic diseases and that the implications of our other reported lncRNAs in these conditions deserve to be further explored.

In our study, groups with DEG were not fully consistent with global expression profiles in preeclamptic and growth-restricted placentas described in the current literature. Omic approaches applied to complex diseases such as PE and IUGR face many challenges, particularly, when using human samples. In this case, the heterogeneity among different populations, sample size, differences between techniques, diversity of platforms, sampling strategies, clinical definitions, and complexity of these syndromes at the clinical and molecular level, play important roles in the lack of consistency of results derived from omic studies [10,37]. Therefore, our results require careful interpretation and warrant further investigation to elucidate the exact contribution of lncRNAs in placental ischemic diseases. Importantly, the data in this study was carefully analyzed using *p*-value correction and a larger sample set for the validation assays. Furthermore, one of the strengths of our study relies on our study groups inclusion criteria that prevented clinical intra-group heterogeneity, by including only PE with severe features, confirmed IUGR with hemodynamic alterations in the fetoplacental circulation and sings of placental insufficiency and all early-onset severe PE for the PE-IUGR, although the particular clinical characteristics of this last group did limit our analysis, and we consider that other strategies should be applied in the study of PE with IUGR to further characterize this condition as it represents the most severe spectrum of the disease and appears to have a particular molecular signature that is not comparable with healthy term placentas. 

Nevertheless, the comparative analysis on the placental transcriptome performed in this study clearly shows that PE and IUGR share altered placental pathophysiological pathways mainly associated with immunological processes, cholesterol, and protein metabolism. Our results also suggest that these conditions are undergoing similar epigenetic regulation regarding lncRNAs expression, although further functional and in silico analysis are needed in order to get deeper insights into the pathways and processes being regulated by these transcripts. 

## 4. Materials and Methods

### 4.1. Ethics Statement

Biological samples and clinical data were collected in the Instituto Nacional de Pertinatología and the Hospital of Ginecología y Obstetricia No. 4, “Luis Castelazo Ayala”, Instituto Mexicano del Seguro Social, in Mexico City, Mexico. This study was approved by the Research and Ethical Committee from the Instituto Nacional de Perinatología in Mexico City, Mexico, reference number IRB00001944; Protocol Number:2230-10101-01-16, March 2016. All participants read and signed a written informed consent prior to enrollment.

### 4.2. Study Groups

All participants had Latino ancestry and resided in Mexico City. Women with a diagnosis of gestational or pre-gestational diabetes, chronic hypertension, cardiomyopathies, immunological and renal diseases, treatment with drugs that could affect metabolism or inflammation during pregnancy, and cases with documented fetal and genetic abnormalities were excluded from the study.

A total of 45 pregnant women with intact membranes, no clinical evidence of intrauterine infection, with a singleton pregnancy, and who delivered by cesarean section (C-section) with no evidence of active labor were included in the study. Four study groups were defined using the following criteria:

(i) Preeclampsia (PE) with severe features diagnosed according to the ACOG 2013 criteria and following the 2019 ACOG practice bulletin guidelines on PE; systolic blood pressure ≥ 160 mmHg and/or diastolic blood pressure ≥ 110 mmHg, and a least one severity criteria (impaired liver function, proteinuria > 300 mg in 24 h, new development of renal insufficiency, thrombocytopenia, cerebral and visual disturbances).
(ii) Intrauterine growth restriction (IUGR) without maternal complications, included cases with fetuses with ultrasound-estimated fetal weight below the 10th percentile, calculated using the Hadlock intrauterine growth curve. All cases presented a history of abnormal doppler waveforms in the venous duct, aortic isthmus, uterine, umbilical, and/or middle cerebral artery in the last ultrasound.
(iii) Severe preeclampsia with Intrauterine growth restriction (PE-IUGR) included cases of women with early-onset severe preeclampsia and diagnosis of intrauterine growth restriction according to the criteria mentioned above.
(iv) Control group was constituted of women with pregnancies at term (>37 weeks of gestation) who delivered normal weight newborns (birthweight between 10th–90th percentile) by elective or iterative C-section, without any major maternal-fetal complications. Follow up was carried out up to six weeks postpartum to discard the development of hypertensive disorders.

### 4.3. Data Collection

Clinical data were collected from maternal and neonatal medical records, including anthropometric and placental histopathology reports.

### 4.4. Sample Collection

Full-thickness placental tissue was collected immediately after C-sections taking samples from the middle region of the placenta, close to the umbilical cord insertion. Right after collection samples were transported to the lab (at 4 °C) and within one hour, samples were rinsed with cold 1× Red Blood Cells lysis buffer and washed with cold 1× PBS to eliminate contamination with maternal blood.

### 4.5. RNA Extraction, Quality, and Integrity Determination.

RNA extraction was performed from 30–100 mg of fresh placental tissue, using RNeasy Fibrous Tissue Mini Kit^®^ (Qiagen, Valencia, CA, USA) following the manufacturer’s instructions. Purity and concentration of total RNA were measured using a NanoDrop^®^ UV-Vis spectrophotometer (Thermo Fisher Scientific Inc., Wilmington, DE, USA). RNA Integrity Number (RIN) was analyzed with the Agilent 2100 Bioanalyzer (Agilent Technologies, Palo Alto, CA, USA). Sampling and RNA extraction was performed by the same personnel using the same methodology.

### 4.6. Microarray Analysis

A one-color microarray-based gene expression analysis was performed using Agilent SurePrint G3 Human Gene Expression v3 8 × 60K Microarrays (G4851C, Agilent Technologies). The glass slides were scanned using an Agilent G2565BA microarray scanner. Relative target intensity was calculated using Agilent Feature Extraction image analysis Software (AFE) version 11.5.1 (Agilent Technologies). Microarray procedures were carried out according to the manufacturer’s instructions by the same personnel using reagents and slides from the same batch. Data array has been deposited in GEO with number GSE147776.

### 4.7. Data Analysis

#### 4.7.1. Microarray Pre-Processing

The AFE software was employed to extract intensity values summarized per probe set from Agilent microarrays. Bioinformatic analysis of microarrays was conducted in Bioconductor within the R computational environment. Initially, non-detected probes, as evaluated by AFE, were removed (i.e., gIsGeneDetected = 0). Subsequently, raw intensity values were Log2 transformed, background adjusted, and quantile normalized using the robust multiarray analysis (RMA) algorithm. Probe-level normalized intensities values were summarized to give single expression level by transcript. Finally, to retain most variability, transcripts in which IQR was below median IQR were filtered out.

#### 4.7.2. Differential Expression Analysis

Normalized and summarized expression data was used as input for the linear models for microarray data analysis algorithm (LIMMA) to assess differential expression of genes between different experimental conditions. False discovery rate adjustment (Benjamini & Hochberg) was employed to correct *p*-values for multiple hypothesis tests. Genes with fold-change > 1.5 and FDR < 0.1 were considered as differentially expressed and selected for further analysis. Pearson correlation analysis was performed using expression levels of selected genes, considering all possible pair-wise comparisons between them. Correlations with *p* < 0.05 were deemed significant.

#### 4.7.3. Functional Enrichment

We used Enrichr program (https://amp.pharm.mssm.edu/Enrichr) to retrieve the most updated version of KEGG_human, GO_Biological_processes, and GO_Molecular_function annotations and performed an over-representation analysis on differentially expressed mRNAs.

### 4.8. Quantitative Real Time PCR

To validate microarray results, ten genes were selected from the top list of differential expression between the study groups and analyzed by quantitative real-time PCR (RT-qPCR). cDNA was synthesized from 1.5 mg of total RNA according to the manufacturer instructions (SuperScript™ III Reverse Transcriptase Invitrogen™, Carlsbad, CA, USA). All RT-qPCR reactions were performed using pre-made TaqMan Gene Expression Assays and the Universal TaqMan gene expression Master Mix (Applied Biosystems, Life technologies, Foster City, CA, USA) (Table S3). The housekeeping gene Glyceraldehyde-3-Phosphate Dehydrogenase (GAPDH) was used in all reactions as endogenous control and negative controls lacked template inputs. Reactions were performed in 96 well plates in CFX-96 Real-Time PCR system (BIO-RAD, Hercules, CA, USA). All genes were analyzed for the differential placental expression in IUGR (*n* = 12), PE (*n* = 11), IUGR-PE (*n* = 11) and normal pregnancy (*n* = 11), and the relative mRNA and lncRNA levels were determined by comparative C_T_ method. 

## Figures and Tables

**Table 1:** Maternal and newborn Characteristics of study groups.

**(a) Individuals for Microarray Analysis**	**IUGR (*n* = 7)**	**PE (*n* = 7)**	**PE-IUGR (*n* = 6)**	**NP (*n* = 8)**
Maternal Age (Years)	26 ± 1.4	30.57 ± 1.5	30.33 ± 2.6	30.57 ± 6.18
pBMI (kg/m^2^)	26.4 ± 1.7	29.9 ± 1.4	25.36 ± 1.66	24.3 ± 0.9
GWG (kg)	7.4 ± 2.4	6.42 ± 1.3	9.5 ± 1.79	8.3 ± 1.6
Nulliparity (%)	50	14	50	0
SBP (mmHg)	108.4 ± 5.1	167.8 ± 3.1 *	171.6 ± 3.8 *	103.5 ± 3.8
DBP (mmHg)	71.14 ± 5.3	112.14 ± 2.85 *	116.6 ± 1.9 *	68 ± 1.75
24 h Urine Protein (mg/dL)	ND	370.03 ± 79.52	1296.8 ± 407.6	ND
Urea (mg/dL)	16.22 ± 2.5	18.95 ± 2.8 *	25.49 ± 4.7	13.2 ± 1.6
Creatinine (mg/dL)	0.51 ± 0.03	0.58 ± 0.39	0.74 ± 0.05 *	0.54 ± 0.41
UA (mg/dL)	4.13 ± 0.9	5.12 ± 0.6 *	5.15 ± 2.3 *	3.55 ± 1.13
Hb (g/dL)	12.88 ± 0.63	13.25 ± 0.45	14.4 ± 0.44 *	12.98 ± 0.36
Platelet count (× 10^9^/L)	240.42 ± 10.26	201.57 ± 17.59	252.83 ± 40.85	286.16 ± 43.91
Early Onset (%)	14	85	100	N/A
Gestational Age at birth (Weeks)	38.5 ± 0.48	32.9 ± 0.98 *	32.01 ± 1.15 *	38.5 ± 0.48
Hadlock Percentile⊥	3.1 ± 0.85	37.2 ± 17.5	3 ± 0.85	N/A
Newborn weight (g)	2175.5 ± 241.3 *	1958 ± 176.79 *	1136.66 ± 176.79 *	3167 ± 130.69
Newborn length (cm)	45 ± 1.75 *	43.71 ± 1.83 *	37.5 ± 1.6 *	49.21 ± 0.63
Fetal Sex (M/F)	4/3	4/3	1/5	5/2
**(b) Individuals for RT-qPCR assays**	**IUGR** **(*n* = 12)**	**PE** **(*n* = 11)**	**PE-IUGR** **(*n* = 11)**	**NP** **(*n* = 11)**
Maternal Age (Years)	26.8 ± 1.8	30.4 ± 1.4	32.2 ± 2.1	32.4 ± 2
pBMI (kg/m^2^)	26 ± 1.26	39.9 ± 1.06	25.9 ± 1.3	27.5 ± 1.2
GWG (kg)	8.19 ± 1.52	6.3 ± 1.3	8.19 ± 1.5	9.6 ± 1.6
Nulliparity (%)	25	9.1	36.3 *	0
SBP (mmHg)	108.8 ± 3.5	167 ± 2.5 *	171.3 ± 3.07 *	105.6 ± 2.7
DBP (mmHg)	72.3 ± 3.6	112.3 ± 1.8 *	113.9 ± 2.7 *	68.8 ± 1.2
24 h Urine Protein (mg/dL)	ND	479.4 ± 132.6	1287.7 ± 279.8	ND
Urea (mg/dL)	18.1 ± 1.8	27.9 ± 7.8 *	24.4 ± 3.3 *	14.2 ± 1.3
Creatinine (mg/dL)	0.51 ± 0.02	0.73 ± 0.9	0.75 ± 0.4 *	0.55 ± 0.2
UA (mg/dL)	3.9 ± 0.8	5.9 ± 0.5 *	5.5 ± 0.57 *	3.8 ± 0.25
Hb (g/dL)	13 ± 0.38	13.3 ± 0.32	14.3 ± 0.3	13.2 ± 0.3
Platelet count (× 10^9^/L)	234.9 ± 14.7	214.3 ± 20.9	258.9 ± 29.1	261.7 ± 26.5
Early Onset (%)	8.3	75	100	N/A
Gestational Age at birth (Weeks)	36.8 ± 0.58 *	33.6 ± 0.87 *	32.3 ± 0.78 *	38.7 ± 0.3
Hadlock Hadlock Percentile⊥	3.1 ± 0.6	28.9 ± 13.2	3 ± 0.5	N/A
Newborn weight (g)	2175.3 ± 144.87 *	2088 ± 163.3 *	1191.7 ± 125.6 *	33175 ± 138.9
Newborn length (cm)	45 ± 1.05 *	43.7 ± 1.3 *	37.9 ± 1.1 *	49.7 ± 0.5
Fetal Sex (M/F)	6/6	6/5	4/7	6/5

Data are given as mean ± SME, unless otherwise stated. ⊥ Hadlock percentile for fetuses at the moment of diagnosis. DBP, Diastolic blood pressure; F, Female; IUGR, Intrauterine growth restriction; GWG, Gestational Weight Gain; Hb, Hemoglobin; M, Male; N/A, Not Applicable; ND, Not determine; NP, Normal Pregnancy; pBMI, Pregestational Body Mass Index; PE, Preeclampsia; PE-IUGR, Preeclampsia and Intrauterine Growth Restriction; SDP, Systolic Blood Pressure; UA, Uric Acid. * *p* value < 0.05 compared to normal pregnancy. Student’s T test (for quantitative Variables), Mann–Whitney U test (for quantitative variables not normally distributed) or X^2^ (For binary variables).

**Table 2:** Top Enrich terms of differentially expressed genes in IUGR.

Index	Term	*p*-Value	Odds Ratio	Genes
**Down-Regulated Genes**				
1	Neuroactive ligand-receptor interaction	0.00743	4.11	*KISS, UTS2R, P2RY6, CCK, RXFP3*
2	Fatty acid biosynthesis	0.04582	21.37	*OLAH*
3	Arginine biosynthesis	0.07297	13.23	*NOS3*
4	Platelet activation	0.07363	4.48	*NOS3, ITGB3*
5	Relaxin signaling pathway	0.07986	4.27	*NOS3, RXFP3*
6	Fluid shear stress and atherosclerosis	0.08952	4.00	*NOS3, ITGB3*
7	PI3K-Akt signaling pathway	0.13524	2.35	*NOS3, ITGB3, SGK2*
8	Ether lipid metabolism	0.15608	5.91	*GDPD1*
9	Arginine and proline metabolism	0.16216	5.67	*NOS3*
10	Proteoglycans in cancer	0.16337	2.76	*ITGB3, ANK3*
11	N-Glycan biosynthesis	0.16519	5.56	*MAN1A2*
12	Regulation of actin cytoskeleton	0.18001	2.60	*ITGB3, SPATA13*
13	VEGF signaling pathway	0.19192	4.71	*NOS3*
**Up-Regulated Genes**				
1	Cytokine-cytokine receptor interaction	0.00000	4.78	*IL10, CCL23, IL15, IL1R2, CCL4L2, PRL, CXCL10, CXCL11, CD40LG, IL18RAP, IFNG, IL1B, CXCR3, IL2RB, TNFRSF8, CCL18, IL17D, CCR2*
2	Chemokine signaling pathway	0.00001	4.93	*CXCL10, ITK, CXCL11, CCL23, SHC3, GNG4, CCL4L2, CXCR3, GNB5, ADCY1, CCL18, CCR2*
3	Primary immunodeficiency	0.00001	12.67	*ZAP70, CD40LG, CD8B, LCK, TAP1, CD3D*
4	Natural killer cell mediated cytotoxicity	0.00001	5.96	*ZAP70, SHC3, IFNG, KIR2DS2, LCK, SH2D1B, GZMB, CD48, KLRC1, ITGAL*
5	Neuroactive ligand-receptor interaction	0.00003	3.47	*OXTR, GZMA, F2R, HTR2B, FPR3 PRL, RXFP1, C3, P2RY8, GAL, CNR1, SST, ADORA3, TAC3, VIP*
6	Hematopoietic cell lineage	0.00003	6.44	*CD8B, CD5, IL1R2, IL1B, CD38, HLA-DQA2, CD3D, CD22*
7	T cell receptor signaling pathway	0.00005	6.19	*IL10, ITK, ZAP70, CD40LG, CD8B, IFNG, LCK, CD3D*
8	Antigen processing and presentation	0.00006	7.10	*IFNG, CD8B, KIR2DS2, TAP1, KLRC1, B2M, HLA-DQA2*
9	Th17 cell differentiation	0.00007	5.84	*ZAP70, IFNG, LCK, IL1B, IL2RB, IL17D, HLA-DQA2, CD3D*
10	Allograft rejection	0.00012	10.28	*IL10, CD40LG, IFNG, GZMB, HLA-DQA2*
11	Graft-versus-host disease	0.00017	9.53	*IFNG, IL1B, GZMB, KLRC1, HLA-DQA2*
13	Th1 and Th2 cell differentiation	0.00018	5.94	*ZAP70, IFNG, LCK, IL2RB, CD3D, RUNX3, HLA-DQA2*

## Supplementary Materials

Supplementary Materials can be found at https://www.mdpi.com/1422-0067/21/10/3597/s1.

## Abbreviations

PE	Preeclampsia
IUGR	Intrauterine Growth Restriction
PE-IUGR	Preeclampsia with Intrauterine Growth Restriction
NP	Normal Pregnancy
pBMI	Pregestational Body Mass Index
GA	Gestational Age
GWG	Gestational Weight Gain
SGA	Small for Gestational Age
LGA	Large for Gestational Age
