# Supplementary material for: Placental Microarray Profiling Reveals Common mRNA and lncRNA Expression Patterns in Preeclampsia and Intrauterine Growth Restriction
Source: Int J Mol Sci. 2020 May 20;21(10):3597. doi: 10.3390/ijms21103597 (PMC7279523; doi:10.3390/ijms21103597)
Supplement: Supplementary file 1 [file ijms-21-03597-s001.pdf]

Supplementary Table S1. Placental Histopathological Findings in Preeclampsia and Intrauterine Growth Restriction.

Placental Histopathology	IUGR %*n*/*n*	PE %*n*/*n*	PE-IUGR %*n*/*n*	*p*		
				IUGR vs. PE	IUGR vs. PE-IUGR	PE vs. PE-IUGR
Distal villous hypoplasia	40 (4/10)	75 (6/8)	100 (3/3)	0.5271	0.7055	0.3173
Placental infarction	80 (8/10)	100 (8/8)	100 (3/3)	1	0.1317	0.1317
Villous hypermaturation patterns	50 (5/10)	87.5 (7/8)	100 (3/3)	0.5637	0.4795	0.2059
Syncytial knots	0 (0/10)	25 (2/8)	66.7 (⅔)	0.1573	0.1573	1
Massive perivillous fibrin deposition	30 (3/10)	25 (2/8)	0 (0/3)	0.6547	0.08326	0.1573

Supplementary Table S2. List of significantly differentially expressed genes by contrast.

Gene	logFC	P.Value	adj.P.Val	Contrasts	Transcript_Type
IGFBP1	6.0	8.25E-06	0.08	PS-CTRL	mRNA
EGFR-AS1	3.7	1.23E-05	0.08	PS-CTRL	lnc-RNA
C11orf86	2.8	3.24E-05	0.09	PS-CTRL	mRNA
STAR	3.6	4.82E-05	0.09	PS-CTRL	mRNA
ITGAD	1.6	4.94E-05	0.09	PS-CTRL	mRNA
FGG	4.8	5.21E-05	0.09	PS-CTRL	mRNA
PBX4	2.3	6.71E-05	0.09	PS-CTRL	mRNA
SOAT2	2.3	6.74E-05	0.09	PS-CTRL	mRNA
ZNF683	2.9	7.43E-05	0.09	PS-CTRL	mRNA
LRRC15	3.8	7.81E-05	0.09	PS-CTRL	mRNA
OLAH	−2.6	8.07E-05	0.09	PS-CTRL	mRNA
CATSPER1	2.4	8.56E-05	0.09	PS-CTRL	mRNA
TWIST2	1.8	9.38E-05	0.09	PS-CTRL	mRNA
TMEM132C	2.2	9.76E-05	0.09	PS-CTRL	mRNA
FLJ31356	2.0	8.39E-05	0.09	PS-CTRL	lnc-RNA
lnc-TCL1B-1	−2.2	0.000148222	0.09	PS-CTRL	lnc-RNA
lnc-MRPS5-1	−2.2	0.0001535	0.09	PS-CTRL	lnc-RNA
GNB5	1.6	0.000116919	0.09	PS-CTRL	mRNA
GNGT1	−2.2	0.000119	0.09	PS-CTRL	mRNA
GNG4	2.5	0.000131396	0.09	PS-CTRL	mRNA
lnc-PPM1D-1	−1.6	0.000170353	0.09	PS−CTRL	lnc−RNA
PRL	4.3	0.000164144	0.09	PS−CTRL	mRNA
TRIM64	3.6	0.000169078	0.09	PS−CTRL	mRNA

ADAD1	−1.3	0.000170575	0.09	PS−CTRL	mRNA
FBXO2	2.3	0.000179233	0.09	PS−CTRL	mRNA
CD40LG	1.9	0.000188632	0.09	PS−CTRL	mRNA
TNFRSF8	2.1	0.000194635	0.09	PS−CTRL	mRNA
LOC729739	3.0	0.000198275	0.09	PS−CTRL	mRNA
FOXL2	3.0	0.000208032	0.09	PS−CTRL	mRNA
STON1−GTF2 A1L	2.0	0.000250919	0.09	PS−CTRL	lnc−RNA
PDE6H	3.0	0.000226665	0.09	PS−CTRL	mRNA
CHST2	1.7	0.00024051	0.09	PS−CTRL	mRNA
CPEB1	1.7	0.000240782	0.09	PS−CTRL	mRNA
IL1R2	3.6	0.000243415	0.09	PS−CTRL	mRNA
MED4−AS1	2.5	0.000245393	0.09	PS−CTRL	lnc−RNA
PRUNE2	2.3	0.000250615	0.09	PS−CTRL	mRNA
lnc−VAPA−1	3.2	0.000219149	0.09	PS−CTRL	lnc−RNA
C8orf31	1.6	0.00025149	0.09	PS−CTRL	mRNA
KCNK12	2.8	0.000260278	0.09	PS−CTRL	mRNA
lnc−TRPM7−1	−1.8	0.000270996	0.09	PS−CTRL	lnc−RNA
CABYR	2.7	2.60E−06	0.04	P.RES−CTRL	mRNA
MCEMP1	3.2	1.12E−05	0.06	P.RES−CTRL	mRNA
LOC338797	−1.5	1.37E−05	0.06	P.RES−CTRL	lnc−RNA
IGFBP1	5.9	1.93E−05	0.07	P.RES−CTRL	mRNA
CPEB1	2.1	1.30E−05	0.04	IUGR−CTRL	mRNA
TMEM132C	2.5	1.46E−05	0.04	IUGR−CTRL	mRNA
TWIST2	2.1	2.15E−05	0.04	IUGR−CTRL	mRNA
FGG	5.0	2.35E−05	0.04	IUGR−CTRL	mRNA
CYP2A7	1.6	2.89E−05	0.04	IUGR−CTRL	mRNA
STAR	3.7	3.21E−05	0.04	IUGR−CTRL	mRNA
LOC101929019	−2.1	3.39E−05	0.04	IUGR−CTRL	lnc−RNA
FBXO2	2.6	3.60E−05	0.04	IUGR−CTRL	mRNA
IGFBP1	5.4	3.63E−05	0.04	IUGR−CTRL	mRNA
CABYR	2.2	3.66E−05	0.04	IUGR−CTRL	mRNA
MS4A12	−2.2	4.26E−05	0.04	IUGR−CTRL	mRNA
TNFRSF8	2.3	4.34E−05	0.04	IUGR−CTRL	mRNA
PDZK1IP1	3.8	4.44E−05	0.04	IUGR−CTRL	mRNA
CD40LG	2.1	4.55E−05	0.04	IUGR−CTRL	mRNA
F2R	2.2	4.66E−05	0.04	IUGR−CTRL	mRNA
GNG4	2.7	4.83E−05	0.04	IUGR−CTRL	mRNA
RFPL4B	2.8	5.12E−05	0.04	IUGR−CTRL	mRNA
ZNF683	3.0	5.16E−05	0.04	IUGR−CTRL	mRNA
lnc−TRMT61B−2	−1.6	5.43E−05	0.04	IUGR−CTRL	lnc−RNA
RUNX3	1.9	5.48E−05	0.04	IUGR−CTRL	mRNA
SAMD3	2.0	6.32E−05	0.04	IUGR−CTRL	mRNA
lnc−F8A2−1	−2.3	6.65E−05	0.04	IUGR−CTRL	lnc−RNA

CYP3A7	1.8	7.24E−05	0.04	IUGR−CTRL	mRNA
PRUNE2	2.5	7.43E−05	0.04	IUGR−CTRL	mRNA
LRRC15	3.8	7.48E−05	0.04	IUGR−CTRL	mRNA
PRL	4.6	7.72E−05	0.04	IUGR−CTRL	mRNA
IL1R2	4.0	7.76E−05	0.04	IUGR−CTRL	mRNA
PCDH11X	−2.0	7.98E−05	0.04	IUGR−CTRL	mRNA
EGFR−AS1	3.2	8.75E−05	0.04	IUGR−CTRL	lnc−RNA
C8orf31	1.7	8.81E−05	0.04	IUGR−CTRL	mRNA
lnc−SPATA9−1	−1.9	0.000103978	0.04	IUGR−CTRL	lnc−RNA
TAC3	3.7	0.000105524	0.04	IUGR−CTRL	mRNA
DAW1	3.3	0.000110116	0.04	IUGR−CTRL	mRNA
IGSF10	3.1	0.000110719	0.04	IUGR−CTRL	mRNA
TM4SF19	1.6	0.000116817	0.04	IUGR−CTRL	mRNA
RNASE4	2.1	0.000118813	0.04	IUGR−CTRL	mRNA
ADAD1	−1.4	0.000119749	0.04	IUGR−CTRL	mRNA
lnc−VAPA−1	3.3	0.000121603	0.04	IUGR−CTRL	lnc−RNA
HTR2B	2.8	0.000126938	0.04	IUGR−CTRL	mRNA
ANKRD22	2.2	0.000132255	0.04	IUGR−CTRL	mRNA
LINC00473	3.7	0.000133217	0.04	IUGR−CTRL	lnc−RNA
lnc−ESCO2−2	1.6	0.000136852	0.04	IUGR−CTRL	lnc−RNA
MGST1	3.7	0.000145938	0.04	IUGR−CTRL	mRNA
DKFZp434J0226	2.3	0.000149307	0.04	IUGR−CTRL	lnc−RNA
lnc−IL1R2−1	3.4	0.000151115	0.04	IUGR−CTRL	lnc−RNA
lnc−AAAS−1	2.1	0.000151284	0.04	IUGR−CTRL	lnc−RNA
OR2AP1	−2.0	0.000156813	0.04	IUGR−CTRL	mRNA
CHST2	1.7	0.000160063	0.04	IUGR−CTRL	mRNA
ESRRG	−1.4	0.000161185	0.04	IUGR−CTRL	mRNA
PRG2	3.3	0.000161779	0.04	IUGR−CTRL	mRNA
lnc−CHADL−2	−1.5	0.000163073	0.04	IUGR−CTRL	lnc−RNA
CD8B	1.9	0.000166495	0.04	IUGR−CTRL	mRNA
EPYC	3.7	0.000169879	0.04	IUGR−CTRL	mRNA
CATSPER1	2.3	0.000176967	0.04	IUGR−CTRL	mRNA
KCNK12	2.9	0.000177811	0.04	IUGR−CTRL	mRNA
lnc−THSD4−2	−2.2	0.000179806	0.04	IUGR−CTRL	lnc−RNA
C11orf86	2.4	0.000182976	0.04	IUGR−CTRL	mRNA
lnc−TCL1B−1	−2.2	0.00018373	0.04	IUGR−CTRL	lnc−RNA
SPATA17	1.8	0.000184485	0.04	IUGR−CTRL	mRNA
lnc−LRTM2−1	−2.5	0.000192404	0.04	IUGR−CTRL	lnc−RNA
GNB5	1.6	0.000193229	0.04	IUGR−CTRL	mRNA
ADCY1	2.2	0.000202628	0.04	IUGR−CTRL	mRNA
PLA2G7	2.3	0.000203042	0.04	IUGR−CTRL	mRNA
CLEC10A	1.6	0.000210211	0.04	IUGR−CTRL	mRNA
HLA−DQA2	2.9	0.000211841	0.04	IUGR−CTRL	mRNA

TRIM64	3.6	0.000212138	0.04	IUGR−CTRL	mRNA
OR5H14	3.0	0.000219904	0.04	IUGR−CTRL	mRNA
MAP3K5	1.4	0.000220729	0.04	IUGR−CTRL	mRNA
CD52	1.6	0.000232085	0.05	IUGR−CTRL	mRNA
XLOC_l2_009139	2.9	0.000238059	0.05	IUGR−CTRL	lnc−RNA
SLC10A1	−1.4	0.000239942	0.05	IUGR−CTRL	mRNA
CHI3L2	2.7	0.000248108	0.05	IUGR−CTRL	mRNA
ALDH1A2	3.3	0.000256783	0.05	IUGR−CTRL	mRNA
CHRDL1	4.3	0.000261357	0.05	IUGR−CTRL	mRNA
IQCA1	1.7	0.000265497	0.05	IUGR−CTRL	mRNA
FOXL2	2.9	0.000275905	0.05	IUGR−CTRL	mRNA
PVRIG	1.7	0.000277524	0.05	IUGR−CTRL	mRNA
GAL	1.9	0.000278683	0.05	IUGR−CTRL	mRNA
ERP27	1.8	0.000282581	0.05	IUGR−CTRL	mRNA
lnc−FAM53A−1	1.7	0.000286837	0.05	IUGR−CTRL	lnc−RNA
CENPV	1.9	0.000303677	0.05	IUGR−CTRL	mRNA
FCRL3	2.0	0.000305278	0.05	IUGR−CTRL	mRNA
LOC338797	−1.2	0.000308691	0.05	IUGR−CTRL	lnc−RNA
lnc−TAF4B−4	−2.0	0.000309577	0.05	IUGR−CTRL	lnc−RNA
F12	2.2	0.000335637	0.05	IUGR−CTRL	mRNA
FOLH1B	2.4	0.000335927	0.05	IUGR−CTRL	mRNA
RBP4	2.8	0.000351099	0.05	IUGR−CTRL	mRNA
IL2RB	2.7	0.000365503	0.05	IUGR−CTRL	mRNA
LOC102723721	3.2	0.000366813	0.05	IUGR−CTRL	uncharacterized
KLK9	−1.6	0.000367122	0.05	IUGR−CTRL	mRNA
RGL4	2.2	0.000369389	0.05	IUGR−CTRL	mRNA
SECISBP2L	−1.3	0.000377502	0.05	IUGR−CTRL	mRNA
SCML4	2.0	0.000378354	0.05	IUGR−CTRL	mRNA
RGS2	1.6	0.000382116	0.05	IUGR−CTRL	mRNA
PDZK1	2.6	0.000388702	0.05	IUGR−CTRL	mRNA
HN1	1.7	0.000390841	0.05	IUGR−CTRL	mRNA
lnc−TNFRSF17−2	−1.4	0.000399423	0.05	IUGR−CTRL	lnc−RNA
DEPDC7	2.1	0.000403639	0.05	IUGR−CTRL	mRNA
TIMP3	1.4	0.000404213	0.05	IUGR−CTRL	mRNA
LOC100131303	−1.9	0.000404918	0.05	IUGR−CTRL	lnc−RNA
SMPD3	1.8	0.000408207	0.05	IUGR−CTRL	mRNA
UTS2R	−2.9	0.000408671	0.05	IUGR−CTRL	mRNA
MCEMP1	2.3	0.000414032	0.05	IUGR−CTRL	mRNA
MAGEB5	−1.5	0.000416586	0.05	IUGR−CTRL	mRNA
lnc−PRKAG2−1	−1.4	0.000424218	0.05	IUGR−CTRL	lnc−RNA
ARSE	1.8	0.000430985	0.05	IUGR−CTRL	mRNA
LIPK	1.3	0.000438196	0.05	IUGR−CTRL	mRNA

PTPN7	1.8	0.000438484	0.05	IUGR−CTRL	mRNA
lnc−INHBA−1	−1.4	0.000446365	0.05	IUGR−CTRL	lnc−RNA
NTN4	1.6	0.000447845	0.05	IUGR−CTRL	mRNA
PDE6H	2.8	0.000449222	0.05	IUGR−CTRL	mRNA
KLRC1	2.1	0.000451959	0.05	IUGR−CTRL	mRNA
ZNF583	1.7	0.000452832	0.05	IUGR−CTRL	mRNA
PBX4	2.0	0.000455867	0.05	IUGR−CTRL	mRNA
MAOB	3.2	0.000456858	0.05	IUGR−CTRL	mRNA
ABLIM2	1.9	0.000457918	0.05	IUGR−CTRL	mRNA
WNT4	2.0	0.000467553	0.05	IUGR−CTRL	mRNA
MIR762HG	1.5	0.00046922	0.05	IUGR−CTRL	lnc−RNA
SMOX	1.7	0.000471587	0.05	IUGR−CTRL	mRNA
GZMB	1.9	0.000479154	0.05	IUGR−CTRL	mRNA
LINC00967	−1.6	0.000479285	0.05	IUGR−CTRL	lnc−RNA
FNDC3A	−1.3	0.000482089	0.05	IUGR−CTRL	mRNA
LOC102725314	−1.6	0.00048742	0.05	IUGR−CTRL	lnc−RNA
MED4−AS1	2.4	0.00048965	0.05	IUGR−CTRL	lnc−RNA
PPP4R4	2.4	0.000491918	0.05	IUGR−CTRL	mRNA
LOC102723906	−1.3	0.000497315	0.05	IUGR−CTRL	uncharacterized
ALOXE3	−1.7	0.000503593	0.05	IUGR−CTRL	mRNA
lnc−OSTF1−1	−1.9	0.000505936	0.05	IUGR−CTRL	lnc−RNA
CISD3	−3.2	0.000518824	0.06	IUGR−CTRL	mRNA
CNR1	3.7	0.000521978	0.06	IUGR−CTRL	mRNA
DNASE1L3	1.4	0.000533908	0.06	IUGR−CTRL	mRNA
XLOC_l2_011737	−2.3	0.000538059	0.06	IUGR−CTRL	lnc−RNA
LOC100129917	−1.6	0.000538602	0.06	IUGR−CTRL	lnc−RNA
LOC100507389	−1.6	0.000548906	0.06	IUGR−CTRL	lnc−RNA
CILP2	2.8	0.0005678	0.06	IUGR−CTRL	mRNA
GNLY	3.2	0.000567926	0.06	IUGR−CTRL	mRNA
CHI3L1	2.1	0.00057166	0.06	IUGR−CTRL	mRNA
PCDH20	3.9	0.000573523	0.06	IUGR−CTRL	mRNA
SKAP1	1.7	0.000581543	0.06	IUGR−CTRL	mRNA
C1orf106	2.1	0.000589736	0.06	IUGR−CTRL	mRNA
SHC3	1.6	0.000595615	0.06	IUGR−CTRL	mRNA
TUBA3D	1.6	0.000608416	0.06	IUGR−CTRL	mRNA
UBD	3.4	0.000614629	0.06	IUGR−CTRL	mRNA
METAP1D	1.7	0.00061935	0.06	IUGR−CTRL	mRNA
lnc−RP11−90J19.1.1−3	−2.2	0.000624171	0.06	IUGR−CTRL	lnc−RNA
FOXL2NB	2.9	0.000624836	0.06	IUGR−CTRL	mRNA
SOD2	1.2	0.000626595	0.06	IUGR−CTRL	mRNA
CYTIP	1.7	0.000630972	0.06	IUGR−CTRL	mRNA
MARCO	1.8	0.0006451	0.06	IUGR−CTRL	mRNA

CD5	2.4	0.000653042	0.06	IUGR−CTRL	mRNA
CLDN16	−1.6	0.000653461	0.06	IUGR−CTRL	mRNA
lnc−CYTL1−1	−1.4	0.000662836	0.06	IUGR−CTRL	lnc−RNA
LGALS13	−1.9	0.000667784	0.06	IUGR−CTRL	mRNA
P4HA3	2.5	0.000684343	0.06	IUGR−CTRL	mRNA
KLRB1	2.0	0.000684973	0.06	IUGR−CTRL	mRNA
LCK	1.8	0.000692208	0.06	IUGR−CTRL	mRNA
HIST1H1E	−1.8	0.000693374	0.06	IUGR−CTRL	mRNA
ADORA3	1.6	0.000707803	0.06	IUGR−CTRL	mRNA
KCNIP4−IT1	−1.2	0.000713975	0.06	IUGR−CTRL	lnc−RNA
lnc−SLC12A8−1	2.1	0.000724688	0.06	IUGR−CTRL	lnc−RNA
lnc−TMEM88B −1	−1.7	0.000733956	0.06	IUGR−CTRL	lnc−RNA
IL17D	1.9	0.000751408	0.06	IUGR−CTRL	mRNA
ELMOD1	1.9	0.000751635	0.06	IUGR−CTRL	mRNA
TAP1	1.2	0.000755757	0.06	IUGR−CTRL	mRNA
ERVV−2	−2.0	0.000766536	0.06	IUGR−CTRL	mRNA
CXCR3	1.3	0.000788742	0.06	IUGR−CTRL	mRNA
ASCL2	2.4	0.000791075	0.06	IUGR−CTRL	mRNA
CCL23	1.9	0.000791543	0.06	IUGR−CTRL	mRNA
LOC101929295	−1.4	0.000804784	0.06	IUGR−CTRL	lnc−RNA
lnc−MRPS5−1	−1.9	0.000804913	0.06	IUGR−CTRL	lnc−RNA
UCHL1	2.8	0.000806829	0.06	IUGR−CTRL	mRNA
AFF1	−1.5	0.000815501	0.06	IUGR−CTRL	mRNA
DIRAS2	2.9	0.000818017	0.06	IUGR−CTRL	mRNA
OLAH	−2.1	0.000818198	0.06	IUGR−CTRL	mRNA
NDP	3.2	0.000822175	0.06	IUGR−CTRL	mRNA
WBSCR17	2.1	0.000823818	0.06	IUGR−CTRL	mRNA
TRIM17	−1.6	0.000825157	0.06	IUGR−CTRL	mRNA
lnc−NUP88−1	−1.3	0.000829021	0.06	IUGR−CTRL	lnc−RNA
ESX1	2.1	0.000833669	0.06	IUGR−CTRL	mRNA
PTPN22	1.6	0.000834372	0.06	IUGR−CTRL	mRNA
GOLGA6L1	−1.3	0.000840164	0.06	IUGR−CTRL	mRNA
CHST15	1.3	0.000842379	0.06	IUGR−CTRL	mRNA
LOC100506384	−1.4	0.000844989	0.06	IUGR−CTRL	lnc−RNA
LGALS14	−1.8	0.000846932	0.06	IUGR−CTRL	mRNA
CXCL10	2.6	0.000849014	0.06	IUGR−CTRL	mRNA
C1QTNF9	2.6	0.000853793	0.06	IUGR−CTRL	mRNA
LRRN4CL	2.8	0.000855967	0.06	IUGR−CTRL	mRNA
CD3D	1.7	0.00087211	0.06	IUGR−CTRL	mRNA
USP30−AS1	1.6	0.000876007	0.06	IUGR−CTRL	lnc−RNA
XLOC_l2_0066 24	−1.1	0.00087704	0.06	IUGR−CTRL	lnc−RNA
LINC01118	−2.1	0.000877208	0.06	IUGR−CTRL	lnc−RNA
GPRC5A	2.2	0.000888466	0.06	IUGR−CTRL	mRNA

lnc−AMMECR1−1	1.3	0.000889212	0.06	IUGR−CTRL	lnc−RNA
CCL18	3.1	0.000894963	0.06	IUGR−CTRL	mRNA
MYO16−AS1	2.5	0.000896026	0.06	IUGR−CTRL	lnc−RNA
KCNJ16	2.3	0.000917952	0.06	IUGR−CTRL	mRNA
SPACA6P−AS	−1.5	0.000918193	0.06	IUGR−CTRL	lnc−RNA
WT1	3.0	0.000921661	0.06	IUGR−CTRL	mRNA
LOC100129345	−2.1	0.000925917	0.06	IUGR−CTRL	lnc−RNA
SOAT2	1.8	0.000953932	0.07	IUGR−CTRL	mRNA
MYBPH	−1.6	0.000965611	0.07	IUGR−CTRL	mRNA
GREB1	1.6	0.000972739	0.07	IUGR−CTRL	mRNA
CD48	2.1	0.000980416	0.07	IUGR−CTRL	mRNA
lnc−EPSTI1−1	1.8	0.000984275	0.07	IUGR−CTRL	lnc−RNA
LINC01449	−1.3	0.000996623	0.07	IUGR−CTRL	lnc−RNA
LSAMP	2.0	0.00099846	0.07	IUGR−CTRL	mRNA
CST7	2.2	0.000999542	0.07	IUGR−CTRL	mRNA
SLAMF7	2.4	0.001005876	0.07	IUGR−CTRL	mRNA
ITGB3	−1.6	0.001023196	0.07	IUGR−CTRL	mRNA
PAWR	−1.4	0.001023248	0.07	IUGR−CTRL	mRNA
SLC1A6	2.6	0.001035931	0.07	IUGR−CTRL	mRNA
LOC255187	−1.4	0.001041037	0.07	IUGR−CTRL	lnc−RNA
GZMA	2.0	0.001058674	0.07	IUGR−CTRL	mRNA
SH2D1B	2.0	0.001075009	0.07	IUGR−CTRL	mRNA
CEND1	1.5	0.001086491	0.07	IUGR−CTRL	mRNA
FOLH1	2.2	0.001088624	0.07	IUGR−CTRL	mRNA
ZBED6	−1.4	0.001090231	0.07	IUGR−CTRL	mRNA
EPDR1	2.1	0.001098599	0.07	IUGR−CTRL	mRNA
S100A4	1.1	0.001113529	0.07	IUGR−CTRL	mRNA
LOC100132874	−2.1	0.001114455	0.07	IUGR−CTRL	mRNA
LOC101926934	2.7	0.001114742	0.07	IUGR−CTRL	uncharacterized
lnc−PPM1D−1	−1.3	0.001123104	0.07	IUGR−CTRL	lnc−RNA
SIGLEC10	1.6	0.001129285	0.07	IUGR−CTRL	mRNA
LAYN	1.2	0.001139114	0.07	IUGR−CTRL	mRNA
lnc−NDUFS5−2	−1.3	0.001144322	0.07	IUGR−CTRL	lnc−RNA
TAGAP	1.4	0.001147801	0.07	IUGR−CTRL	mRNA
ERV3−1	−2.1	0.0011482	0.07	IUGR−CTRL	mRNA
TTR	3.5	0.001161967	0.07	IUGR−CTRL	mRNA
STAB2	2.8	0.001164541	0.07	IUGR−CTRL	mRNA
NKG7	1.7	0.001175342	0.07	IUGR−CTRL	mRNA
lnc−RABEPK−1	−1.5	0.001177415	0.07	IUGR−CTRL	lnc−RNA
DAPP1	1.4	0.001199205	0.07	IUGR−CTRL	mRNA
TCL6	−1.7	0.001203249	0.07	IUGR−CTRL	lnc−RNA
HCN2	−3.0	0.001226324	0.07	IUGR−CTRL	mRNA
lnc−CEBPB−4	−1.3	0.001228666	0.07	IUGR−CTRL	lnc−RNA

CSDC2	1.7	0.001229097	0.07	IUGR−CTRL	mRNA
SCARNA1	−2.0	0.001239199	0.07	IUGR−CTRL	other
LOC729739	2.5	0.001245851	0.07	IUGR−CTRL	mRNA
RBP1	1.6	0.001249699	0.07	IUGR−CTRL	mRNA
AKNA	−1.2	0.001263587	0.07	IUGR−CTRL	other
CCDC103	1.3	0.00126561	0.07	IUGR−CTRL	mRNA
TMEM63B	−2.2	0.00126689	0.07	IUGR−CTRL	mRNA
GLT1D1	1.7	0.001270943	0.07	IUGR−CTRL	mRNA
GAS1	2.0	0.001279032	0.07	IUGR−CTRL	mRNA
STON1−GTF2A1L	1.7	0.001279075	0.07	IUGR−CTRL	lnc−RNA
IL15	1.8	0.001303353	0.07	IUGR−CTRL	mRNA
KIR2DS2	2.0	0.001324277	0.07	IUGR−CTRL	mRNA
SIT1	1.6	0.001332386	0.07	IUGR−CTRL	mRNA
LINC00598	−1.5	0.001332667	0.07	IUGR−CTRL	lnc−RNA
CD69	2.3	0.001340674	0.07	IUGR−CTRL	mRNA
LAG3	1.9	0.001375772	0.08	IUGR−CTRL	mRNA
PGR	1.6	0.001389317	0.08	IUGR−CTRL	mRNA
lnc−PHKB−5	−2.1	0.001414276	0.08	IUGR−CTRL	lnc−RNA
TMEM27	2.0	0.001417013	0.08	IUGR−CTRL	mRNA
GDAP1	1.4	0.001421656	0.08	IUGR−CTRL	mRNA
SNORA2B	−1.6	0.001426957	0.08	IUGR−CTRL	snRNA
SGK2	−1.5	0.001433966	0.08	IUGR−CTRL	mRNA
CD6	1.7	0.001436698	0.08	IUGR−CTRL	mRNA
ITGAD	1.2	0.001437449	0.08	IUGR−CTRL	mRNA
ANK3	−1.4	0.001448583	0.08	IUGR−CTRL	mRNA
LOC100507283	1.5	0.001455299	0.08	IUGR−CTRL	lnc−RNA
AADAC	3.4	0.001457791	0.08	IUGR−CTRL	mRNA
UNC5B−AS1	1.3	0.00145837	0.08	IUGR−CTRL	lnc−RNA
DEFB103A	−1.5	0.001462051	0.08	IUGR−CTRL	mRNA
FAM110C	3.0	0.00147239	0.08	IUGR−CTRL	mRNA
CRYM	−1.5	0.001475482	0.08	IUGR−CTRL	mRNA
MXRA7	1.0	0.001477888	0.08	IUGR−CTRL	mRNA
lnc−SOCS5−2	−1.4	0.001489554	0.08	IUGR−CTRL	lnc−RNA
IL1B	2.4	0.001491734	0.08	IUGR−CTRL	mRNA
KCND2	2.4	0.001498729	0.08	IUGR−CTRL	mRNA
LCMT1−AS1	−1.4	0.001502805	0.08	IUGR−CTRL	lnc−RNA
RXFP1	2.4	0.001504898	0.08	IUGR−CTRL	mRNA
lnc−RBM22−1	−2.3	0.001512069	0.08	IUGR−CTRL	lnc−RNA
POTEB3	−1.8	0.001514042	0.08	IUGR−CTRL	mRNA
LOC100129935	−1.6	0.00153108	0.08	IUGR−CTRL	pseudogene
MAN1A2	−1.5	0.001550061	0.08	IUGR−CTRL	mRNA
CCK	−3.2	0.001557754	0.08	IUGR−CTRL	mRNA
lnc−LGALS14−1	−1.4	0.001588349	0.08	IUGR−CTRL	lnc−RNA

KDELR3	1.3	0.001613918	0.08	IUGR−CTRL	mRNA
ZNF217	−1.3	0.001617839	0.08	IUGR−CTRL	mRNA
XLOC_l2_009136	−1.1	0.001622694	0.08	IUGR−CTRL	lnc−RNA
ANTXR1	1.3	0.001623093	0.08	IUGR−CTRL	mRNA
TIFAB	2.0	0.001627319	0.08	IUGR−CTRL	mRNA
SIGLEC17P	1.9	0.001642548	0.08	IUGR−CTRL	mRNA
P2RY6	−1.2	0.001644485	0.08	IUGR−CTRL	mRNA
USP43	−1.5	0.001645638	0.08	IUGR−CTRL	mRNA
LOC100289120	−1.3	0.001649035	0.08	IUGR−CTRL	mRNA
HAMP	1.3	0.001657842	0.08	IUGR−CTRL	mRNA
lnc−FURIN−1	−1.1	0.001681577	0.08	IUGR−CTRL	lnc−RNA
TRAF3IP3	1.2	0.001685608	0.08	IUGR−CTRL	mRNA
FAM131B	1.2	0.001710149	0.08	IUGR−CTRL	mRNA
lnc−IKZF2−1	−1.1	0.001713223	0.08	IUGR−CTRL	lnc−RNA
lnc−KLF10−1	−2.0	0.001715422	0.08	IUGR−CTRL	lnc−RNA
lnc−SULF2−3	−1.4	0.001720239	0.08	IUGR−CTRL	lnc−RNA
FHOD3	1.9	0.001728642	0.08	IUGR−CTRL	mRNA
lnc−SOD2−2	1.4	0.001735663	0.08	IUGR−CTRL	lnc−RNA
LINC00482	−1.1	0.00173744	0.08	IUGR−CTRL	lnc−RNA
ITK	1.8	0.001751737	0.08	IUGR−CTRL	mRNA
MB	1.4	0.001752163	0.08	IUGR−CTRL	mRNA
LINC01338	1.7	0.001758866	0.08	IUGR−CTRL	lnc−RNA
lnc−DMXL2−1	−2.2	0.001761488	0.08	IUGR−CTRL	lnc−RNA
PLIN1	1.8	0.001773563	0.08	IUGR−CTRL	mRNA
FPR3	1.5	0.001793447	0.08	IUGR−CTRL	mRNA
ALDH1A1	1.8	0.001823675	0.08	IUGR−CTRL	mRNA
GDPD1	−1.6	0.001823988	0.08	IUGR−CTRL	mRNA
EGLN3	2.1	0.001825048	0.08	IUGR−CTRL	mRNA
FAM159B	2.1	0.001829728	0.08	IUGR−CTRL	mRNA
CXCR2P1	1.8	0.001843508	0.08	IUGR−CTRL	pseudogene
ANKRD30BP3	−1.4	0.001876517	0.08	IUGR−CTRL	mRNA
HSD11B1	1.7	0.00188097	0.08	IUGR−CTRL	mRNA
SMC5−AS1	−1.2	0.001960651	0.09	IUGR−CTRL	lnc−RNA
GPR45	−1.9	0.001963083	0.09	IUGR−CTRL	mRNA
NEBL−AS1	−1.3	0.001978769	0.09	IUGR−CTRL	lnc−RNA
SPOCK1	2.9	0.00198205	0.09	IUGR−CTRL	mRNA
PAEP	2.8	0.001983921	0.09	IUGR−CTRL	mRNA
LOC100293612	−1.6	0.001992719	0.09	IUGR−CTRL	uncharacterized
lnc−GLDN−2	−1.5	0.001994009	0.09	IUGR−CTRL	lnc−RNA
GSTM1	1.0	0.002001027	0.09	IUGR−CTRL	mRNA
SLPI	2.5	0.002010674	0.09	IUGR−CTRL	mRNA
XLOC_l2_010328	1.4	0.00201087	0.09	IUGR−CTRL	lnc−RNA

lnc−FITM2−1	−1.4	0.002018341	0.09	IUGR−CTRL	lnc−RNA
ANKRD20A5P	−1.5	0.002036634	0.09	IUGR−CTRL	pseudogene
PARP9	1.1	0.002039687	0.09	IUGR−CTRL	mRNA
LOC100507195	1.6	0.002053712	0.09	IUGR−CTRL	lnc−RNA
RNF224	1.3	0.002059453	0.09	IUGR−CTRL	mRNA
LOC101929633	−1.2	0.002066796	0.09	IUGR−CTRL	lnc−RNA
lnc−PLCD3−1	−2.2	0.002068612	0.09	IUGR−CTRL	lnc−RNA
lnc−FOLR3−1	−1.4	0.00206957	0.09	IUGR−CTRL	lnc−RNA
DUSP26	1.3	0.00207924	0.09	IUGR−CTRL	mRNA
FAM105A	1.2	0.002082458	0.09	IUGR−CTRL	mRNA
SPATA13	−1.0	0.002083583	0.09	IUGR−CTRL	mRNA
CXCL11	2.5	0.002092788	0.09	IUGR−CTRL	mRNA
MEP1A	2.2	0.002096283	0.09	IUGR−CTRL	mRNA
TMEM200C	−2.4	0.002128741	0.09	IUGR−CTRL	mRNA
LOC100287042	−1.2	0.002147064	0.09	IUGR−CTRL	lnc−RNA
CCR2	2.0	0.002147389	0.09	IUGR−CTRL	mRNA
SNORA49	−1.7	0.002155563	0.09	IUGR−CTRL	snRNA
CCDC177	−1.8	0.002166611	0.09	IUGR−CTRL	mRNA
FGF11	1.3	0.002169067	0.09	IUGR−CTRL	mRNA
NOS3	−1.3	0.002179362	0.09	IUGR−CTRL	mRNA
SLC22A14	−1.8	0.002188382	0.09	IUGR−CTRL	mRNA
PRDM1	1.7	0.002206612	0.09	IUGR−CTRL	mRNA
lnc−AC074091.13.1−1	−1.9	0.002213982	0.09	IUGR−CTRL	lnc−RNA
CD300LF	1.7	0.002215276	0.09	IUGR−CTRL	mRNA
ZAP70	1.6	0.002222449	0.09	IUGR−CTRL	mRNA
ENTPD3	2.8	0.002226258	0.09	IUGR−CTRL	mRNA
LAIR2	2.7	0.002227507	0.09	IUGR−CTRL	mRNA
SLC43A2	−1.3	0.002228054	0.09	IUGR−CTRL	mRNA
lnc−EIF2AK3−4	−1.9	0.00223469	0.09	IUGR−CTRL	lnc−RNA
TMEM45A	1.3	0.002244344	0.09	IUGR−CTRL	mRNA
KRTAP11−1	−1.7	0.002248062	0.09	IUGR−CTRL	mRNA
lnc−ARAP2−2	−1.9	0.002295618	0.09	IUGR−CTRL	lnc−RNA
P2RY8	1.3	0.002300866	0.09	IUGR−CTRL	mRNA
SLC25A30	1.2	0.002304581	0.09	IUGR−CTRL	mRNA
LOC101929353	−1.3	0.002305833	0.09	IUGR−CTRL	lnc−RNA
LINC00426	1.7	0.002305894	0.09	IUGR−CTRL	lnc−RNA
lnc−EMP2−2	−1.8	0.0023278	0.09	IUGR−CTRL	lnc−RNA
CAPG	1.7	0.002334617	0.09	IUGR−CTRL	mRNA
LGALS8−AS1	−1.6	0.002342077	0.09	IUGR−CTRL	lnc−RNA
LINC01119	−1.4	0.002345152	0.09	IUGR−CTRL	lnc−RNA
XLOC_l2_000407	−1.5	0.002375401	0.09	IUGR−CTRL	lnc−RNA
lnc−PSD4−1	−1.5	0.002386767	0.09	IUGR−CTRL	lnc−RNA
LOC101927910	−1.5	0.002395792	0.09	IUGR−CTRL	mRNA

ASMT	−1.3	0.002399832	0.09	IUGR−CTRL	lnc−RNA
LOC100130238	−1.5	0.002403614	0.09	IUGR−CTRL	lnc−RNA
LOC101929531	1.8	0.002414208	0.09	IUGR−CTRL	uncharacterized
lnc−S1PR2−1	−1.3	0.002425734	0.09	IUGR−CTRL	lnc−RNA
GPR18	1.9	0.002434741	0.09	IUGR−CTRL	mRNA
C3	2.1	0.002454063	0.09	IUGR−CTRL	mRNA
OXTR	2.4	0.002471123	0.09	IUGR−CTRL	mRNA
FAM171B	−1.8	0.002502632	0.09	IUGR−CTRL	mRNA
B2M	1.1	0.002503243	0.09	IUGR−CTRL	mRNA
lnc−C17orf101−1	−1.4	0.002506567	0.09	IUGR−CTRL	lnc−RNA
FLJ31356	1.5	0.002510017	0.09	IUGR−CTRL	lnc−RNA
LY6K	1.4	0.00251149	0.09	IUGR−CTRL	mRNA
S100A3	1.8	0.00251151	0.09	IUGR−CTRL	mRNA
XLOC_l2_003882	−1.4	0.002520177	0.09	IUGR−CTRL	lnc−RNA
IL18RAP	1.2	0.00252108	0.09	IUGR−CTRL	mRNA
lnc−C11orf30−1	1.3	0.002531953	0.09	IUGR−CTRL	lnc−RNA
EXTL1	1.7	0.002533799	0.09	IUGR−CTRL	mRNA
lnc−ARID2−2	−1.2	0.002536546	0.09	IUGR−CTRL	lnc−RNA
MYOZ1	1.2	0.00253745	0.09	IUGR−CTRL	mRNA
lnc−TMEM132B−4	−1.8	0.002564931	0.09	IUGR−CTRL	lnc−RNA
XLOC_l2_013462	−1.1	0.002566989	0.09	IUGR−CTRL	lnc−RNA
LOC283352	1.2	0.002574658	0.09	IUGR−CTRL	lnc−RNA
BAGE	−1.5	0.002579074	0.09	IUGR−CTRL	mRNA
TMPRSS4−AS1	−3.0	0.002601474	0.09	IUGR−CTRL	lnc−RNA
CTNND2	1.4	0.002604725	0.09	IUGR−CTRL	mRNA
PSPHP1	3.5	0.002613761	0.09	IUGR−CTRL	mRNA
LINC01424	−1.4	0.002622775	0.09	IUGR−CTRL	lnc−RNA
LINC00606	−1.0	0.002629449	0.09	IUGR−CTRL	lnc−RNA
lnc−RAD23B−10	−1.3	0.002635396	0.09	IUGR−CTRL	lnc−RNA
CILP	2.1	0.002636355	0.09	IUGR−CTRL	mRNA
lnc−ZNF85−1	−1.7	0.00265202	0.09	IUGR−CTRL	lnc−RNA
LOC100132735	1.4	0.002652574	0.09	IUGR−CTRL	lnc−RNA
IFNG	2.2	0.002666473	0.09	IUGR−CTRL	mRNA
DLK2	1.8	0.002680001	0.09	IUGR−CTRL	mRNA
CCL4L2	1.7	0.002684629	0.09	IUGR−CTRL	mRNA
DPP6	1.2	0.002684878	0.09	IUGR−CTRL	mRNA
ASMTL−AS1	−1.0	0.002728594	0.09	IUGR−CTRL	lnc−RNA
LOC101929241	−1.4	0.002754342	0.09	IUGR−CTRL	lnc−RNA
C1orf105	1.5	0.002756583	0.09	IUGR−CTRL	mRNA
IKZF3	1.7	0.002761962	0.09	IUGR−CTRL	mRNA

LINC00924	1.3	0.002763319	0.09	IUGR−CTRL	lnc−RNA
XLOC_l2_014694	−1.2	0.002763983	0.09	IUGR−CTRL	lnc−RNA
PGM2L1	1.1	0.002782011	0.09	IUGR−CTRL	mRNA
HAGLROS	1.6	0.00278251	0.09	IUGR−CTRL	lnc−RNA
SNCAIP	1.4	0.002806348	0.09	IUGR−CTRL	mRNA
ITGAL	1.0	0.00283814	0.09	IUGR−CTRL	mRNA
ITGB7	1.2	0.002857445	0.10	IUGR−CTRL	mRNA
CD22	1.9	0.002859201	0.10	IUGR−CTRL	mRNA
lnc−ZBED1−1	−1.1	0.002864223	0.10	IUGR−CTRL	lnc−RNA
lnc−C1QTNF9−3	−1.5	0.002870511	0.10	IUGR−CTRL	lnc−RNA
HOXD−AS2	2.1	0.002895047	0.10	IUGR−CTRL	lnc−RNA
SERPINA3	2.7	0.002920307	0.10	IUGR−CTRL	mRNA
CPB1	1.3	0.002931426	0.10	IUGR−CTRL	mRNA
KLK10	−1.2	0.002949725	0.10	IUGR−CTRL	mRNA
lnc−MRPS33−1	−2.1	0.002954702	0.10	IUGR−CTRL	lnc−RNA
CHD5	−1.2	0.002955036	0.10	IUGR−CTRL	mRNA
lnc−SPARCL1−1	−1.2	0.002958211	0.10	IUGR−CTRL	lnc−RNA
CTSW	1.7	0.002962898	0.10	IUGR−CTRL	mRNA
ANKRD6	1.3	0.002976426	0.10	IUGR−CTRL	mRNA
SH3D21	−1.2	0.002979674	0.10	IUGR−CTRL	mRNA
IL10	1.2	0.002995702	0.10	IUGR−CTRL	mRNA
ADAMTS6	−1.8	0.002996714	0.10	IUGR−CTRL	mRNA
lnc−TRPM7−1	−1.4	0.003004223	0.10	IUGR−CTRL	lnc−RNA
RXFP3	−1.4	0.003006926	0.10	IUGR−CTRL	mRNA
ZNF354C	−1.1	0.003012467	0.10	IUGR−CTRL	mRNA
ULK4P3	−1.2	0.003035326	0.10	IUGR−CTRL	pseudogene
ULK4	2.2	0.003053565	0.10	IUGR−CTRL	mRNA
VIP	1.2	0.003068752	0.10	IUGR−CTRL	mRNA
CLEC19A	−1.2	0.00308394	0.10	IUGR−CTRL	lnc−RNA
LINC00892	1.3	0.00308566	0.10	IUGR−CTRL	lnc−RNA
LOC100128551	−1.2	0.0030887	0.10	IUGR−CTRL	other
SLC12A8	2.6	0.003101781	0.10	IUGR−CTRL	mRNA
CD38	2.1	0.003104344	0.10	IUGR−CTRL	mRNA
AQP9	1.5	0.003107788	0.10	IUGR−CTRL	mRNA
lnc−PDE4D−1	−1.6	0.003114914	0.10	IUGR−CTRL	lnc−RNA
lnc−ABCD3−1	1.8	0.00311772	0.10	IUGR−CTRL	lnc−RNA
C12orf42	1.8	0.003119082	0.10	IUGR−CTRL	mRNA
KISS1	−2.6	0.003149994	0.10	IUGR−CTRL	mRNA
LOC101928015	1.1	0.003159992	0.10	IUGR−CTRL	uncharacterized
SLC45A4	−1.1	0.003162301	0.10	IUGR−CTRL	lnc−RNA
LOC102724593	1.3	0.003164993	0.10	IUGR−CTRL	lnc−RNA

LOC729680	1.6	0.003189533	0.10	IUGR−CTRL	other
ADAMTS2	1.1	0.003198315	0.10	IUGR−CTRL	mRNA
SST	1.7	0.003217733	0.10	IUGR−CTRL	mRNA
DEPTOR	1.1	0.003218237	0.10	IUGR−CTRL	mRNA
RAB27B	1.1	0.003232296	0.10	IUGR−CTRL	mRNA
FRA10AC1	1.0	0.003241445	0.10	IUGR−CTRL	mRNA
lnc−C22orf26−4	−1.5	0.003245601	0.10	IUGR−CTRL	lnc−RNA
lnc−CACNB3−1	−1.4	0.003248979	0.10	IUGR−CTRL	lnc−RNA
TREM2	1.3	0.003249867	0.10	IUGR−CTRL	mRNA
FXYD3	−1.1	0.003251272	0.10	IUGR−CTRL	mRNA
lnc−FAM27D1.1−3	−1.1	0.003281659	0.10	IUGR−CTRL	lnc−RNA
LOC100505938	−1.4	0.003284798	0.10	IUGR−CTRL	lnc−RNA
SRD5A1	1.2	0.00331183	0.10	IUGR−CTRL	mRNA
C1QTNF9B	1.3	0.003333059	0.10	IUGR−CTRL	mRNA
lnc−EIF3M−2	1.4	0.003340305	0.10	IUGR−CTRL	lnc−RNA
lnc−VCX−2	−1.3	0.003352679	0.10	IUGR−CTRL	lnc−RNA
VSTM2L	1.9	0.003377065	0.10	IUGR−CTRL	mRNA
PSPHP1	6.1	7.01E−06	0.10	P.RES−PS	mRNA
PSPHP1	7.4	1.21E−07	0.00	IUGR−PS	mRNA
PSPH	2.3	2.26E−06	0.02	IUGR−PS	mRNA

Supplementary Table S3. TaqMan Probe Sets Used in the RT−qPCR experiments.

Gene	Gene Name	Taqman Assay
IGFBP1	Insulin Like Growth Factor Binding Protein 1	Hs00236877_m1
FGG	Fibrinogen Gamma Chain	Hs00241037_m1
FBXO2	F−Box Protein 2	Hs00201792_m1
EGFR−AS1	non−coding RNA−	Hs04332527_m1
CPEB1	Cytoplasmic Polyadenylation Element Binding Protein 1	Hs00952081_m1
CHST2	Carbohydrate Sulfotransferase 2	Hs00358839_g1
CD40LG	CD40 Ligand	Hs00163934_m1
CATSPER1	Cation Channel Sperm Associated 1	Hs00364950_m1
CABYR	Calcium Binding Tyrosine Phosphorylation Regulated	Hs00971710_g1
STAR	Steroidogenic Acute Regulatory Protein	Hs00986559_g1

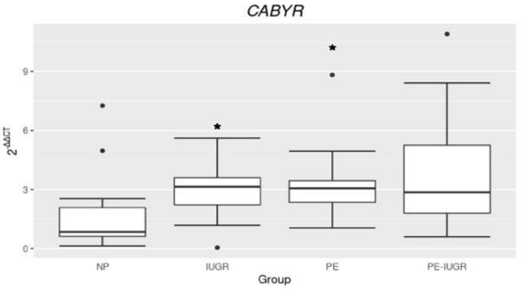

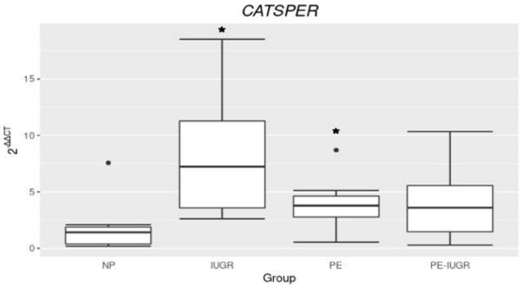

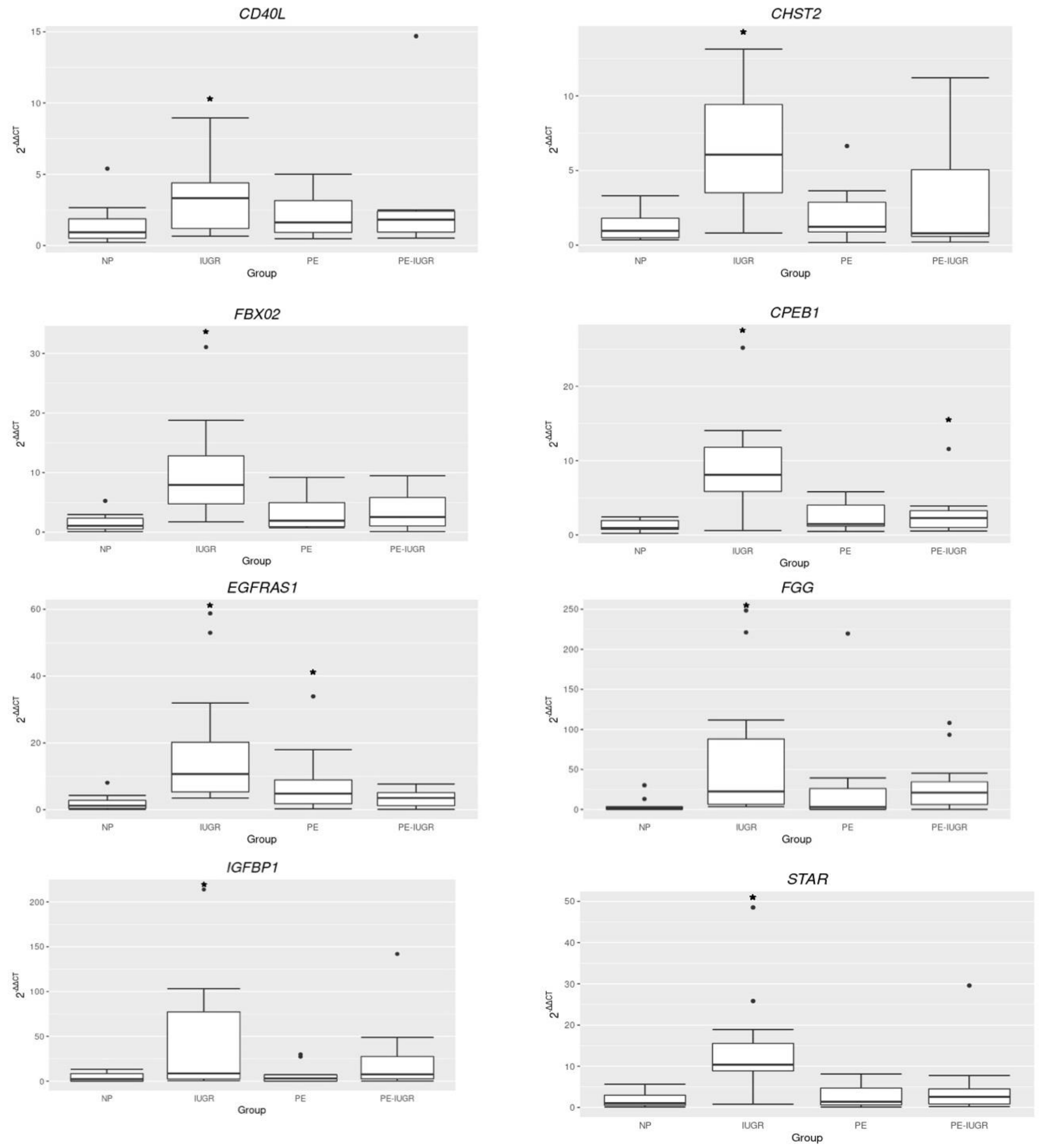

Supplementary Figure S1. Expression profiles of the selected differentially expressed genes from the RT−qPCR assays Boxplots are based on the $2^{-\Delta\Delta Ct}$ values across all studied groups representing normal pregnancies (NP; *n* = 11), Intrauterine Growth Restriction (IUGR; *n* = 12), PE (Preeclampsia; n = 11) and PE−IUGR (Preeclampsia and Intrauterine Growth Restriction; *n* = 11). CATSPER: Cation Channel Sperm Associated 1, CABYR: Calcium Binding Tyrosine Phosphorylation Regulated), CPEB1: Cytoplasmic Polyadenylation Element Binding Protein 1,CD40L CD40 ligand, CHST2: Carbohydrate Sulfotransferase 2, FBX02: F−Box Protein 2, EGFR−AS1: EGFR Antisense RNA 1, FGG: Fibrinogen Gamma Chain, STAR: Fibrinogen Gamma Chain, IGFBP1: Insulin Like Growth Factor Binding Protein 1.
